# Intracellular *Staphylococcus aureus* persisters upon antibiotic exposure

**DOI:** 10.1038/s41467-020-15966-7

**Published:** 2020-05-04

**Authors:** Frédéric Peyrusson, Hugo Varet, Tiep Khac Nguyen, Rachel Legendre, Odile Sismeiro, Jean-Yves Coppée, Christiane Wolz, Tanel Tenson, Françoise Van Bambeke

**Affiliations:** 1 Pharmacologie cellulaire et moléculaire, Louvain Drug Research Institute, Université catholique de Louvain (UCLouvain), Brussels, Belgium; 2 Hub de Bioinformatique et Biostatistique – Département Biologie Computationnelle, Institut Pasteur, USR 3756 CNRS, Paris, France; 3 Institut Pasteur, Plate-forme Transcriptome et Epigenome, Biomics, Centre de Ressources et Recherches Technologiques (C2RT), Paris, France; 4 Institut für Medizinische Mikrobiologie und Hygiene, Tübingen, Germany; 5 Institute of Technology, University of Tartu, Tartu, Estonia

**Keywords:** Antibiotics, Antibacterial drug resistance, Cellular microbiology, Pathogens

## Abstract

Bacterial persister cells are phenotypic variants that exhibit a transient non-growing state and antibiotic tolerance. Here, we provide in vitro evidence of *Staphylococcus aureus* persisters within infected host cells. We show that the bacteria surviving antibiotic treatment within host cells are persisters, displaying biphasic killing and reaching a uniformly non-responsive, non-dividing state when followed at the single-cell level. This phenotype is stable but reversible upon antibiotic removal. Intracellular *S. aureus* persisters remain metabolically active but display an altered transcriptomic profile consistent with activation of stress responses, including the stringent response as well as cell wall stress, SOS and heat shock responses. These changes are associated with multidrug tolerance after exposure to a single antibiotic. We hypothesize that intracellular *S. aureus* persisters may constitute a reservoir for relapsing infection and could contribute to therapeutic failures.

## Abstract (web summary)

Bacterial persister cells exhibit a transient non-growing state and antibiotic tolerance. Here, Peyrusson et al. provide evidence of metabolically active *Staphylococcus aureus* persisters within infected host cells exposed to antibiotics and analyse transcriptomic alterations associated with persistence.

## Introduction

Persisters are subpopulations of cells in bacterial cultures that adopt a transient phenotype characterized by a non-growing state and a tolerance to lethal concentrations of antibiotics^1^. As opposed to resistance, persistence is not genetically inherited. Experimentally, persisters are usually evidenced by biphasic kill curves, when a bulk of susceptible bacteria is rapidly killed by exposure to high antibiotic concentrations, while a small proportion survives for longer time^2,3^. Recent advances in single-cell analyses have allowed characterizing persisters in more details, and notably revealed their non-growing state. There is now convincing and convergent experimental evidence of their clinical relevance, as they contribute to the establishment of chronic infections as well as to the emergence of antibiotic resistance^4^.

The switch to persister phenotype has been largely related to the activation of the stringent response, a global and widely distributed adaptation program that occurs in response to various stresses and modulates many physiological activities. However, its exact role in persistence regulation is still debated^5,6^. Additionally, tolerance is often considered as a passive consequence of growth arrest^7^, which is now challenged by mounting evidence of active responses in some species^8^. Similarly, whether or not dormancy is sufficient to explain antibiotic tolerance is also questioned^9^.

Although persister cells have been identified in all major pathogens^10–13^, a switch to a persister phenotype has been proposed to occur inside eukaryotic cells for only very few intracellular bacteria^14,15^ in response to the environmental stress imposed by the host cell.

Intracellular survival of *Staphylococcus aureus* is widely recognized as a major factor in the recurrence of infections^16^ and intracellular forms of *S. aureus* have been shown to become less responsive to antibiotic action^17^, suggesting a switch to a persister phenotype.

In the present work, we provide evidence for the presence of *S. aureus* intracellular persisters after antibiotic exposure and characterize their dynamics using a fluorescence dilution-based method to monitor bacterial division at the single cell level. We show that intracellular bacterial populations are characterized by a biphasic killing, accompanied by a rapid switch to a uniformly non-dividing and non-responsive state, which is readily reversible upon antibiotic removal. As a potential issue in therapeutic failures, we then aim to better understand the factors leading to antibiotic persistence and tolerance. Using RNA-sequencing we show that these persisters harbor a major transcriptomic reprogramming and remain metabolically active despite prolonged persistence within the host cells. While neither ATP nor amino acid limitation occur, we find that bacteria adjust their central carbon metabolism and redirect transcription to the benefit of a network of adaptive responses. Strikingly, after exposure to a single antibiotic, these responses lead to tolerance to multiple antibiotic classes that act on unrelated targets.

## Results

### *S. aureus* surviving to antibiotics in cells are persisters

**Fig. 1:**
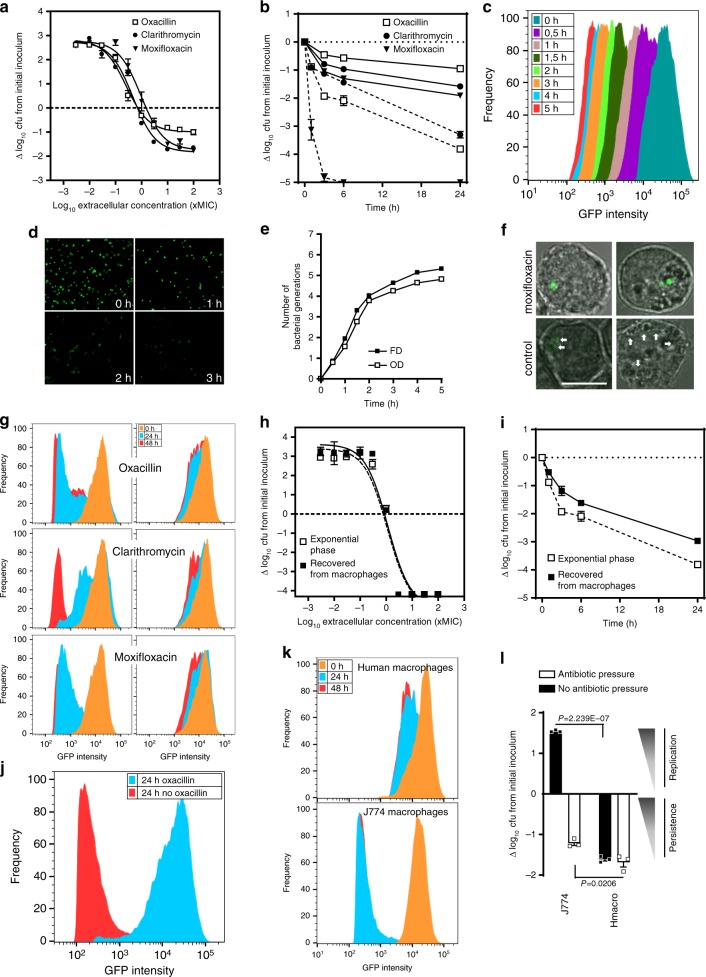
Evidence and dynamics of intracellular persisters of *S. aureus*. **a** Antibiotic activity against *S. aureus* infecting J774 macrophages exposed to increasing concentrations of antibiotics for 24 h (data expressed as log_10_ cfu reduction from postphagocytosis inoculum). **b** Time-kill curves against *S. aureus* infecting J774 macrophages (solid lines) or in exponential phase culture (dotted lines) exposed to 50x MIC of antibiotics for the indicated periods. **c** Fluorescence dilution (FD) experiment with *S. aureus* expressing inducible GFP. Bacteria washed from inducer at the entry of exponential phase were grown in fresh broth. The graph shows flow cytometric profiles of the frequency of events as a function of GFP intensity over time. **d** Corresponding images in epifluorescence microscopy. **e** Corresponding bacterial replication curves determined by FD and OD_620nm_ (OD), which displayed similar doubling times (e.g., 27 min and 28.7 min between 1 h and 2 h, respectively; *N* [number of generations]). **f** Confocal microscopy of infected J774 macrophages exposed to 50x MIC moxifloxacin or under control conditions (2x MIC gentamicin) for 24 h. Arrows: bacteria with diluted signal (bar: 10 μm). **g** Flow cytometric profiles of bacteria recovered from macrophages exposed to 2×(left) or 50x MIC (right) of each antibiotic for the indicated periods. **h**, **i** Activity of oxacillin (**h**, concentration-effect at 24 h; **i**, time-kill curve with 50x MIC oxacillin) in broth, against an exponential phase culture (open symbols) or bacteria recovered from macrophages exposed to 50x MIC oxacillin for 24 h (closed symbols). **j** Flow cytometric profiles of bacteria recovered from macrophages exposed to 50x MIC oxacillin for 24 h (blue), then washed from oxacillin and reincubated in control conditions (2× MIC gentamicin) for an additional period of 24 h (red). **k** Flow cytometric profiles of bacteria recovered from control (2x MIC gentamicin) J774 and human macrophages for the indicated periods. **l** Intracellular inoculum in infected J774 and human macrophages incubated for 24 h with 50× MIC oxacillin or in control conditions (2× MIC gentamicin). Statistical significance was determined by two-tailed Student’s t-test. Data are means ± SEM (**a**, **b**, **h, i, l**) or representatives results (**c, d, e, f, g, j, k**) of three independent experiments. **a, b, e, h, i, l**, Source data are provided as a Source Data file.

Concentration-response curves of typical antistaphylococcal antibiotics targeting the cell wall (oxacillin), protein synthesis (clarithromycin), and replication (moxifloxacin), revealed their inability to clear bacteria from J774 macrophages: after 24 h of infection with high antibiotic concentrations, an antibiotic-tolerant pool of cultivable *S. aureus* persisted inside the macrophages (Fig. 1a). In parallel, time-kill curves performed in the presence of high concentration of each of these antibiotics revealed a biphasic killing: a bulk of the bacterial population was susceptible and rapidly killed while a subpopulation with a slower killing rate was persisting for a much longer period of time. A similar profile was observed against planktonic cultures, but the persisting subpopulation was considerably lower than intracellularly (Fig. 1b). This profile is considered as a hallmark of antibiotic persistence^2,3^.

Persisters are subpopulations that transiently adopt a non-growing and a tolerant state. To confirm these observations and further characterize this phenotype, we first set out to provide evidence for non-growing phenotype of intracellular *S. aureus* by setting up a fluorescence dilution-based method to monitor bacterial division at the single cell level^18^. We used *S. aureus* SH1000 expressing GFP from a tetracycline-inducible promotor, which allows one to follow cell division by monitoring the decrease in GFP signal intensity per cell after removal of the inducer. To validate this approach, bacteria in broth were induced overnight for GFP production, washed from inducer, and diluted to entry into exponential phase, after which their fluorescence signals were analyzed by flow cytometry (Fig. 1c). A homogeneous replication within the bacterial population was observed with unimodal distribution of the signal gradually declining over time, as confirmed in microscopy (Fig. 1d). When comparing the fluorescence dilution with the cfu counting, both methods revealed similar growth curves for 5 generations and similar doubling times (Fig. 1e), validating fluorescence dilution for measurement of bacterial replication^19^.

The same method was applied to characterize the dynamics of intracellular *S. aureus* replication. Macrophages infected by GFP-expressing inoculum revealed bacteria with apparent normal morphology and distinct fluorescence status depending on the antibiotic pressure (Fig. 1f).

This was examined by analyzing the flow cytometry profiles of the replication of intraphagocytic bacteria challenged with different antibiotic concentrations during 48 h of infection (Fig. 1g). Exposure to low antibiotic pressure resulted in an equilibrium between killing and replication. Among the bulk of growing bacteria, a subpopulation rapidly entered into a non-growing state (Supplementary Fig. 1a, b). High antibiotic pressure resulted in both killing of replicating bacteria and larger amounts of non-growing bacteria, leaving a homogenous population of non-growing persisters until the end of the experiment.

Persisters revert to a normal phenotype once antibiotic pressure is removed. We therefore determined the reversibility of the phenotype after antibiotic removal. To this effect, we tested growth resumption and susceptibility towards antibiotics within a non-replicating population collected by cell sorting from infected macrophages after reinoculation in broth (Fig. 1h). A full reversion of the phenotype was observed, in terms of both resulting growth at 24 h and antibiotic susceptibility. Additionally, a biphasic killing profile was also observed for bacteria harvested from macrophages (Fig. 1i). This transient bidirectional switch demonstrates that these intracellular non-dividing subpopulations are persisters. Reversion was also confirmed intracellularly, where persisters started dividing spontaneously within the cell after removal of the antibiotic pressure (Fig. 1j and Supplementary Fig. 1c, d).

This intracellular persistence, together with the ability to resume intracellular replication, are considered as two key determinants for relapsing infections and likely contribute to the clinical observation of rapid recolonization soon after the end of therapy^20^. Intracellular replication is largely described in nonprofessional phagocytes, while intracellular persistence has been reported, although not systematically, in professional phagocytes^21^, and referred to as cell induced-persistence^22^. Yet, intrinsic cell defense mechanisms are critical to trigger either persistence or replication. Illustrating this duality, we observed active bacterial replication in untreated J774 macrophages but not in primary human macrophages (Fig. 1k, l and Supplementary Fig. 1e, f), which can host a viable persister pool, albeit less abundant than the one induced by antibiotic pressure in J774 macrophages.

Thus, we propose here a model in which antibiotic pressure could represent a major trigger factor for intracellular persistence in more permissive cell types. Similar experiments were performed in a series of human cells (epithelial cells, monocytes, osteoblasts, keratinocytes) and confirm the general character of these observations (Supplementary Fig. 2). Because these antibiotic-induced persisters readily reverse upon drug removal, they could constitute a viable reservoir that acts as a major source of dissemination and relapsing infections.

To better understand the factors underlying persistence, we undertook an in-depth RNA-sequencing analysis of these intracellular persisters induced by prolonged antibiotic exposure within eukaryotic cells, as a model for environmental stresses that bacteria face in a clinical context.

### Persisters exhibit an altered transcriptomic profile

**Fig. 2:**
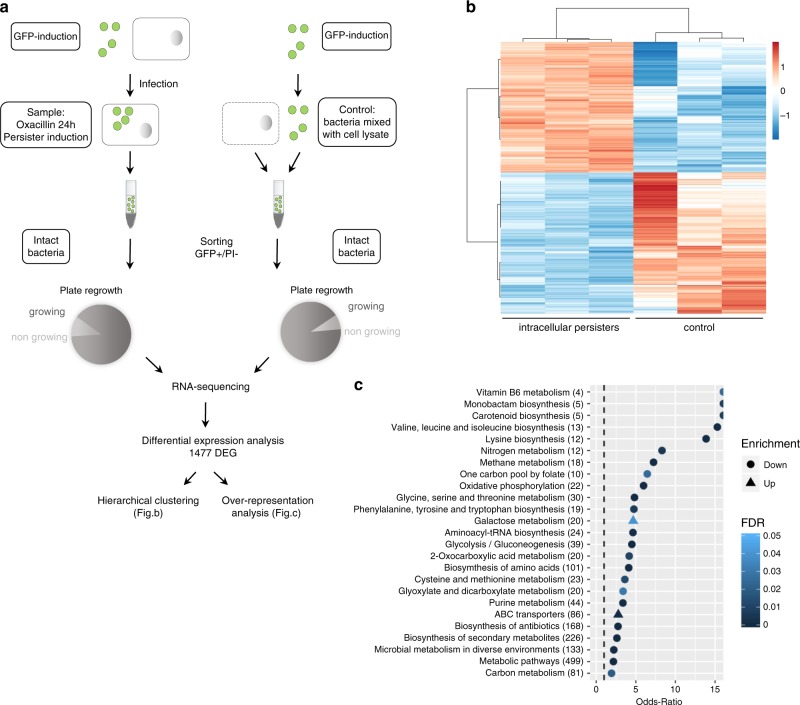
Intracellular antibiotic-induced persisters exhibit a profoundly altered transcriptomic profile. **a** Experimental procedure for sorting and RNA-sequencing of *S. aureus* persisters of SH1000. Cells infected by GFP-expressing bacteria were exposed to 50x MIC oxacillin to allow for the induction of a homogeneous population of persisters. The subset of intact persisters and control samples were collected and sorted by fluorescence-activated cell sorting (FACS) by gating GFP positive (GFP+) and propidium iodide negative signals (PI−), and processed for RNA sequencing (three replicates per condition). 89% and 92% of sorted bacteria from samples and control were able to form colonies, respectively. Differentially expressed genes (DEG) were then analyzed by hierarchical clustering and over-representation analysis. **b** Heatmap displaying hierarchical clustering of DEGs between intracellular persisters and control samples from three biological replicates (color code is function of a Variance Stabilizing Transformation [VST]). **c** Over-representation analysis of DEGs. The graph displays over-represented up- and downregulated KEGG gene-sets (EnrichmentBrowser R package), using the Fisher’s Exact Test and evaluated through Odds-Ratio. Only gene-sets with a false discovery rate (FDR) lower than 0.05 were considered significantly enriched. Numbers in brackets represent the number of genes in the gene-set.

Macrophages were infected by GFP-expressing bacteria and challenged by oxacillin for 24 h to induce homogeneous populations of persisters. Cell sorting was used to isolate the subset of GFP-expressing bacteria (GFP+) that display a propidium iodide negative signal (PI−) (Fig. 2a). The vast majority of GFP+/PI− events were able to form colonies, confirming that they were viable.

The transcriptomic profile of sorted persisters was assessed by RNA-sequencing and reads of each sample were mapped on 2967 protein-coding genes from the reference genome^23^. Differential expression analysis identified 1477 differentially expressed genes (DEG) between the intracellular persisters and control bacteria (Fig. 2b). Hierarchical clustering of this sample set revealed both major divergences between these conditions and high within-group reproducibility. Of the 1477 DEGs, 710 were upregulated and 767 downregulated in persisters (Supplementary Fig. 3).

Over-representation analysis of DEGs revealed that the vast majority of significantly enriched functions corresponds to enrichments in downregulated genes (Fig. 2c). A large group of those belongs to metabolism processes, indicating an overall decrease in metabolic activities, some of them being typically associated with proliferation processes (e.g., nucleotide metabolism and oxidative phosphorylation). Of interest, proliferation-related genes are described to be repressed to the benefit of genes required for stress-defense mechanisms in persisters^24^. Downregulated gene-sets also unveil an important enrichment in metabolism of amino acids (e.g., valine, leucine, isoleucine and lysine biosynthesis), as well as in aminoacyl-tRNA synthetases. Conversely, regarding enrichment in upregulated functions, galactose metabolism was the most significantly enriched function, which may point to deep metabolic network alterations.

### Stringent response contributes to the persistence switch

The persister phenotype has been largely related to the activation of the stringent response (SR)^25^. In response to diverse stresses (including starvation signals and antibiotics), SR is mediated by the rapid synthesis of the alarmones (p)ppGpp, leading to deep transcriptomic reprogramming, especially the repression of proliferation-related genes and the activation of stress resistance- and starvation survival-related genes and a halt of bacterial division^24^. However, recent reports challenge its exact role as a central regulator of persistence^26,27^.

In most firmicutes, (p)ppGpp is synthesized from the GDP/GTP pool via three enzymes: the bifunctional enzyme Rsh that possesses synthase and hydrolase domains, and RelP and RelQ, which only have a synthase domain^24^. The molecular responses initiated by (p)ppGpp seem to differ among species. In some firmicutes, (p)ppGpp has been proposed to affect transcription indirectly through a reduction in the intracellular pool of nucleotides following (p)ppGpp synthesis^28^. Interestingly, the expression of regulators differs depending on the nature of the stress: while Rsh is mainly induced under amino acid limitation^29^, RelP and RelQ have been shown to be mostly induced by cell wall-targeting antimicrobials^30^. In *S. aureus*, CodY regulon is also an integral part of SR: under amino acid starvation, silenced genes are mainly downregulated through the inhibitory effect of (p)ppGpp whereas the majority of activated genes are indirectly regulated via de-repression of CodY. Yet, the implication of SR in intracellular persistence has not yet been conclusively clarified.

**Fig. 3:**
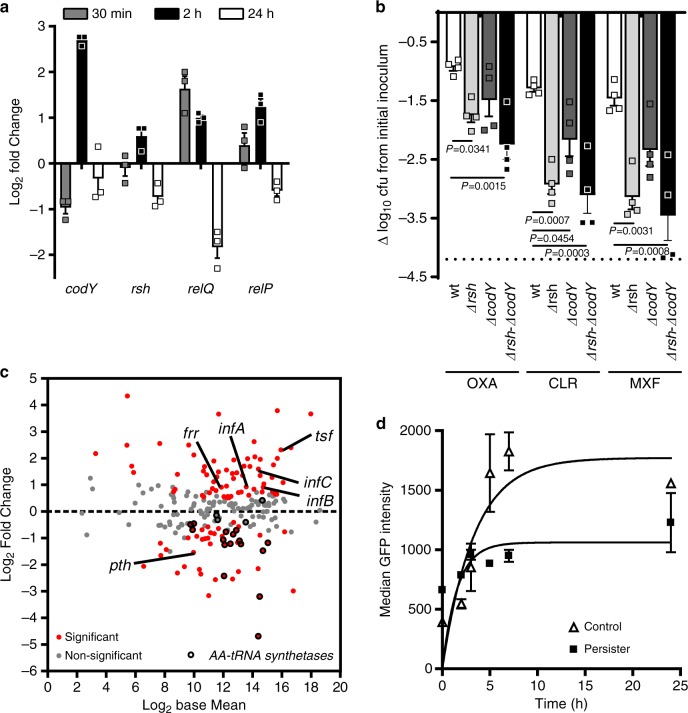
A transitory boost of stringent response contributes to initiate the switch to intracellular persistence. **a** Quantitative real-time PCR of transcripts of stringent response regulators in intracellular persisters exposed to 50× MIC oxacillin for the indicated times. Data, expressed in fold change *vs* control samples (extracellular bacteria mixed with J774 cells lysate), are means ± SEM of three independent experiments. **b** Cfus recovered from macrophages infected by HG001 (WT) and its isogenic mutants and exposed to 50× MIC oxacillin, clarithromycin or moxifloxacin for 24 h. Data (expressed as reduction from the original inoculum) are means ± SEM of four independent experiments. The dotted line indicates the limit of detection. Statistical significance was determined by one-way ANOVA with Dunnett’s post-test. Oxacillin [OXA], clarithromycin [CLR], moxifloxacin [MXF]. **c** MA-plot of genes related to translation^78^. The graph displays the log_2_ Fold Change expression as a function of log_2_ Base Mean (mean expression signal across all samples). Typical members of the function are pointed and aminoacyl-tRNA synthetases are shown in black. The dotted line indicates the basal expression level in control samples. Statistical significance is based on adjusted *P*-value. **d** Rate of GFP synthesis in intracellular *S. aureus*. Macrophages were infected by non-induced bacteria for 24 h, with (persisters) or without (control) 50× MIC oxacillin, and then induced for GFP expression for the indicated periods. Data are means ± SEM of GFP signal from flow cytometric profiles from three independent experiments. **a**, **b**, **d**, Source data are provided as a Source Data file.

Because SR is a highly dynamic process, we investigated the expression of its regulatory network during infection. Quantitative RT-PCR indicated a rapid and transitory boost of these regulators soon after uptake of *S. aureus* by macrophages (Fig. 3a): *relQ* reached its maximal transcription level 30 min after phagocytosis, and *relP, codY*, and, to a lesser extent *rsh*, after 2 h. Limited expression of *rsh* is compatible with its pivotal role in the SR: due to its dual hydrolase/synthase activity, Rsh finely balances the basal levels of (p)ppGpp and prevents its toxic accumulation^31^.

Conversely, at later time points, our transcriptomic approach indicates that the vast majority (73%) of genes within the SR stimulon display a divergent expression signature (repression or non-statistically significant changes; Supplementary Fig. 4), further supporting that the activation of SR is transitory.

To delineate the contribution of SR to the observed phenotype, macrophages were infected with mutants defective in the *rsh* synthase domain and/or *codY* (HG001 strain and isogenic mutants; see Supplementary Table 1) and exposed to high antibiotic pressure (Fig. 3b). Interestingly, with all antibiotics tested, the load of persisters was lower in the *rsh*-negative background than in the parental strain, the difference being less marked with oxacillin. Double mutation in SR regulators led to an additional decrease in the residual load of persisters with oxacillin, and reached maximal effect upon moxifloxacin exposure.

These results strongly suggest that SR is transiently implicated during the initiation of intracellular persistence through (p)ppGpp regulations, in a variable manner depending on the drug exposure. This supports the hypothesis that the central regulation is not entirely dependent on SR and that multiple pathways contribute to the persister phenotype.

### Persisters display dysregulated but active protein synthesis

Originally, persisters were described as being in a strictly dormant state, in which SR shuts down energy-consuming processes by turning translation off, thereby leading to antibiotic tolerance^32^.

In our study, the transcriptomic profile of translation-related genes reveals a dysregulated pattern (Fig. 3c). Indeed, typical members of the protein synthesis machinery were found activated, and displayed a similar trend throughout the whole duration of infection (Supplementary Fig. 5). In line with enrichment analysis, aminoacyl-tRNA synthetase encoding genes were, by contrast, deeply silenced, as is typically encountered under stringent conditions. Additionally, the observed activation of ribosome recycling factor (*frr*) has been described during abortive initiation mechanism (peptidyl drop-off), leading to ribosome reuse^33^.

This trend points to a deep reorientation in protein synthesis rather than a general shutdown, and likely explains the dual expression pattern observed here, in which a part of the translation remains active while another seems to undergo a massive arrest.

To check for the functionality of protein synthesis, we measured the neo-synthesis of GFP as an indication of the translation rate. Intracellular persisters selected by oxacillin responded to induction by producing GFP, indicating that persisters display reduced, but active translation (Fig. 3d). These results led us to conclude that persisters are still metabolically active and that inhibition of translation is not sufficient to explain the antibiotic tolerance of intracellular *S. aureus*.

### Persistence is not triggered by ATP or amino acid limitation

Persistence has been extensively studied in nutrient-poor models, mostly amino acid deprivation, in which bacteria tend to inhibit protein synthesis and promote amino acid biosynthesis. In stationary planktonic cultures, where bacteria are usually observed as dormant, ATP limitation has also been proposed to induce persistence^34^. Recently, stimulation of production of reactive oxygen species by macrophages has been shown to reduce ATP levels and to increase antibiotic tolerance^35^.

**Fig. 4:**
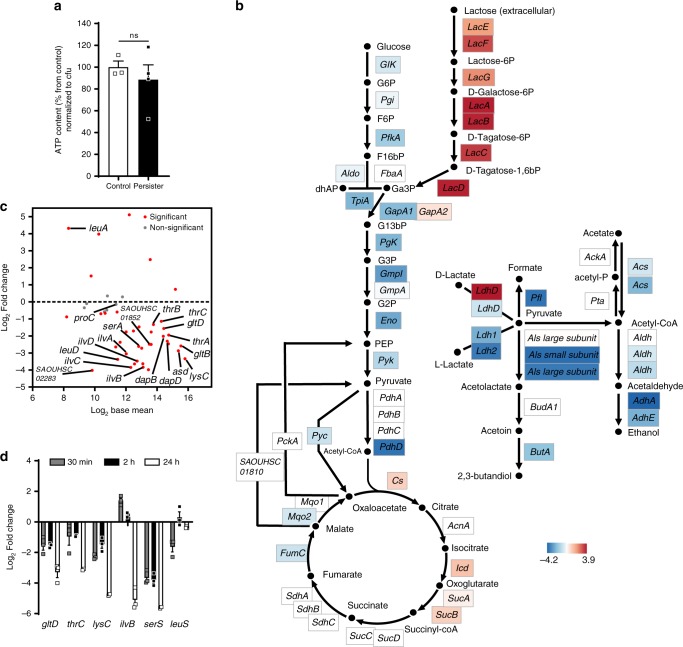
Intracellular persister formation is not triggered by amino acid limitation nor ATP depletion. **a** Intrabacterial ATP concentration in intracellular persisters (exposed to 50× MIC oxacillin for 24 h) and control samples (extracellular bacteria mixed with J774 cells lysate). Appropriate controls were performed to ensure the absence of contamination by eukaryotic ATP. Data are means ± SEM of three independent experiments. Statistical significance was determined by two-tailed Student’s *t*-test. ns non-statistically significant. **b** Schematic pathway of genes related to central carbon metabolism, annotated according to KEGG orthology^78^ and Genbank database^23^ and their log_2_ Fold Change expression levels. **c** MA-plot of genes related to amino acid metabolism within the stringent response stimulon (Supplementary Fig. 4; see reference^55^). The graph displays the log_2_ Fold Change expression as a function of log_2_ Base Mean (mean expression signal across all samples). Genes prominently activated after amino acid depletion^24^ are pointed. The dotted line indicates the basal expression level in control samples. Statistical significance is based on adjusted *P-*value. **d** Quantitative real-time PCR of transcripts of genes related to amino acid synthesis in intracellular persisters exposed to 50x MIC oxacillin for the indicated periods. Data are means ± SEM of three independent experiments. **a**, **d** Source data are provided as a Source Data file.

We therefore measured ATP content in intracellular persisters released from macrophages, and found no significant difference from control samples (Fig. 4a), in line with a study showing that ATP content is not decisive for persister formation in *S. aureus* stationary cultures^36^. These data suggest that intracellular persisters are metabolically active and have adapted their metabolism for ATP maintenance.

The expression pattern of the central metabolic flux (Fig. 4b) reveals metabolic network alterations: we found altered transcripts levels for glycolysis-related enzymes and evidence of an important carbon source shift between glucose and lactose, described as a trigger factor for persistence^37^. This result is in agreement with those of Traxler et al.^38^ who showed that *E. coli* persisters could be formed through a glucose-lactose diauxie in nutrient-rich conditions. Intracellular persisters also drastically adapt their respiration status, with an expression program resembling that observed in anaerobiosis. As revealed by enrichment analysis, genes related to oxidative phosphorylation (Supplementary Fig. 6) were mostly repressed^39^, to the benefit of massive D-lactate fermentation. As the expression levels of TCA cycle genes were rather maintained under these conditions, they could contribute to the redox balance for sustained fermentation.

The transcription signature of the genes related to amino acid metabolism within the SR stimulon (Fig. 4c) revealed that the vast majority were silent after 24 h, and also at earlier infection stages (Fig. 4d), when the transcription of SR regulators is already taking place. We conclude that amino acid limitation is not a trigger factor for intracellular persistence. Because these persisters sustain protein synthesis, we may suppose bacteria rely on other resource pools available in host cell, thereby ruling out the vacuolar nutrient-poor model^40,41^.

Although coherent with studies in Gram-negative pathogens^42^, these conclusions are still under debate for *S. aureus*, for which only amino acid limitation has been proposed as a trigger factor for intracellular persistence^24^.

### Redundant adaptive responses lead to multidrug tolerance

It was previously hypothesized that a central growth arrest would lead to the inactivation of antibiotic targets and to tolerance. Such corrupted targets would prevent fluoroquinolones from generating DNA breaks, aminoglycosides from causing protein mistranslation, or β-lactams from impairing peptidoglycan reticulation^42^. Yet, our model denies pure dormancy of persisters but rather argues for a switch, partly initiated by SR, to a state where active processes ensure functional bacterial maintenance.

**Fig. 5:**
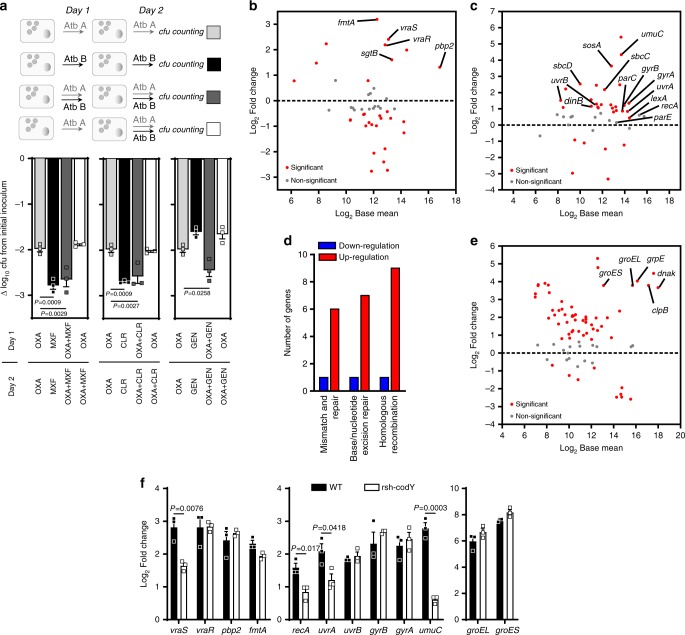
A mosaic of redundant adaptive responses leads to multidrug tolerance. **a** Activity of antibiotic combinations added simultaneously or in succession against intracellular persisters. Intracellular persisters were challenged to 50× MIC of antibiotics alone or in combination, and recovered from macrophages and proceeded for cfu counting after 48 h, following the experimental procedure described above. For combinations, antibiotics were added either at the same time as oxacillin or 24 h after oxacillin. Data (expressed as cfu reduction from the original inoculum) are means ± SEM of three independent experiments. Statistical significance was determined by one-way ANOVA with Dunnett’s post-test. Oxacillin [OXA], moxifloxacin [MXF], clarithromycin [CLR], gentamicin [GEN]. **b**, **c** MA-plots of genes related to peptidoglycan biosynthesis^78^ (extended to *vraS/R* and cell-envelope biogenesis genes from the cell wall stress stimulon)^80^ and SOS response stimulon^46,55^, respectively. The graphs display the log_2_ Fold Change expression as a function of log_2_ Base Mean (mean expression signal across all samples). Typical members of the stimulons are pointed. The dotted lines indicate the basal expression level in control samples. Statistical significance is based on adjusted *P-*value. **d** Number of up- or downregulated DEGs related to DNA repair^78^. **e** MA-plot of genes related to heat shock stimulon^55^. **f** Quantitative real-time PCR of transcripts of determinants of CWSS, SOS response, and heat shock stimulon, from left to right, in HG001 (WT) or HG001 *rsh*-*codY* double mutants (Δ*rsh*-Δ*codY*) exposed to 50× MIC oxacillin for 2 h of infection. Data are means ± SEM of three independent experiments. Statistical significance was determined by two-tailed Student’s *t*-test. **a**, **f** Source data are provided as a Source Data file.

To better understand factors that underlie tolerance, we first examined how persisters induced by one drug would reply to another one. To this effect, intracellular persisters induced by oxacillin for 24 h were then exposed to fluoroquinolones, macrolides or aminoglycosides for an additional 24 h period in the continuing presence of oxacillin (Fig. 5a). When combined with oxacillin, all three antibiotics led to higher reductions in persister counts than oxacillin during the entire duration of the experiment and the kinetics of killing remained biphasic (Supplementary Fig. 7). Strikingly, no additional decrease was observed when the second drug was added *after* exposure to oxacillin. This indicates that oxacillin was able to induce a phenotype conferring a general tolerance to the four antibiotic classes, irrespective of their mechanism of action.

This observation strongly suggests that persister and tolerant phenotype occurs through multiple protective mechanisms intracellularly.

The gene expression of peptidoglycan biosynthesis is significantly reprogrammed within intracellular persisters (Fig. 5b), matching with an activation of the cell wall stress stimulon (CWSS), a protective response to cell wall defects and cell wall-active antibiotics^43^. These changes include the induction of genes within the core of the CWSS: the two-component system *vraS/vraR*, and genes involved in the late steps of peptidoglycan synthesis (i.e., *pbp2*, the transglycosylase *sgtB*, and *fmtA* encoding a penicillin binding protein with low affinity to β-lactams). CWSS induction provides a certain level of tolerance to most VraS/R-inducing agents^43^. Thus, intracellular persisters exhibit active responses for cell wall maintenance that likely mediate the observed tolerance to oxacillin.

Intracellular persisters also elicit an extensive program for preserving genome integrity through the SOS response. This network is a highly conserved DNA damage repair system which has been shown to induce tolerance to fluoroquinolones^44^ and confer protection against the bactericidal effects of β-lactams in *E. coli*^45^. In this pathway, RecA both facilitates recombinational repair and stimulates auto-cleavage of the repressor LexA, resulting in de-repression of genes involved in DNA repair or recombination^46^.

Within intracellular persisters, typical effectors of the SOS network, such as the excision repair systems or mismatch and repair systems, were largely transcribed (Fig. 5c, d) together with genes encoding fluoroquinolone targets, which may cooperatively contribute to the observed fluoroquinolone tolerance.

*S. aureus* persisters also undergo a massive transcription of the heat shock stimulon (Fig. 5e), a central response in stress tolerance. Under our conditions, *dnaK*, *groESL*, and *grpE* transcripts belong to the 98^th^ percentile of the fold change distribution in the transcriptome, thus indicating a drastic activation of this response. The DnaK and GroESL chaperone/chaperonin sequentially function as a crucial bacterial protein folding machinery and participate in the degradation of defective proteins^47^. In the context of multiple stresses and dysregulated translation, its implication in bacterial tolerance is wide. By dealing with damaged proteins, this system is known to participate in tolerance to both β-lactams^48^ and aminoglycosides^49^ and possibly influences the action of macrolides^50^. Macrolides target protein synthesis and cause early peptidyl-tRNA drop-off^51^. Although the mechanisms of macrolide tolerance are largely unknown, a role of this stimulon is conceivable since GroESL has been shown to positively affect the peptidyl-tRNA processing by peptidyl-tRNA hydrolase (Pth)^33^. Additionally, because the uptake of aminoglycosides requires proton motive force^52^, the deeply impaired transcription of the electron transport chain may favorably contribute to the tolerance to aminoglycosides, together with the decrease in translation rate, which has been proposed as a factor leading to tolerance towards protein synthesis inhibitors^53^ (Supplementary Table 2 for summary). Interestingly, this set of adaptive responses redundantly occurs in all permissive host cells tested (Supplementary Fig. 8).

To help clarify how this network of signaling pathways is taking place, and especially their relationship with SR, we studied the effect of *rsh-codY* mutation (HG001 strain and its isogenic mutant) on key determinants of CWSS, SOS and heat shock responses after 2 h of infection, i.e., a time point where SR was active (Fig. 5f). We found that SR positively modulated the expression pattern of SOS response, and, to a lesser extent, of CWSS through *VraS*. Alternatively, the expression of relP/relQ synthetases has been shown to be impacted by the VraS/R system^30^. These mutations, nevertheless, did not annihilate the expression of these genes, and had no effect on heat shock chaperones, leading us to conclude that the SOS response and CWSS are positively modulated but not dependent on SR.

These findings are consistent with overlaps observed in transcription signatures between SR and CWSS or SOS stimulons. Indeed, *relP* has also been described as a core member of CWSS^54^ and conversely, *fmtA* and *sgtB*, as members of SR stimulon, corroborating the entanglements in these responses. Similarly, *lexA* regulator belongs to SR stimulon^55^.

Additionally, in the absence of antibiotic pressure, intracellular bacteria displayed similar inductions of most of the determinants of SOS and heat shock responses, and a basal induction of CWSS, which becomes markedly induced upon antibiotic exposure (Supplementary Fig. 9), suggesting that stresses imposed by the intracellular environment also contribute to the changes observed in these signaling networks.

## Discussion

This work clearly demonstrates the presence of intracellular persisters of *S. aureus* during antibiotic exposure, especially by monitoring bacterial division at a single cell level. We show that antibiotic pressure allows for the induction of homogenous living and non-dividing populations of persisters and that this phenotype is stable but highly reversible upon drug removal. Our model thus indicates a very dynamic bidirectional switch to intracellular persistence. The intracellular environment, by allowing both persistence and replication, could then represent both a privileged reservoir for the pathogen and a major source of relapses after antibiotic removal.

**Fig. 6:**
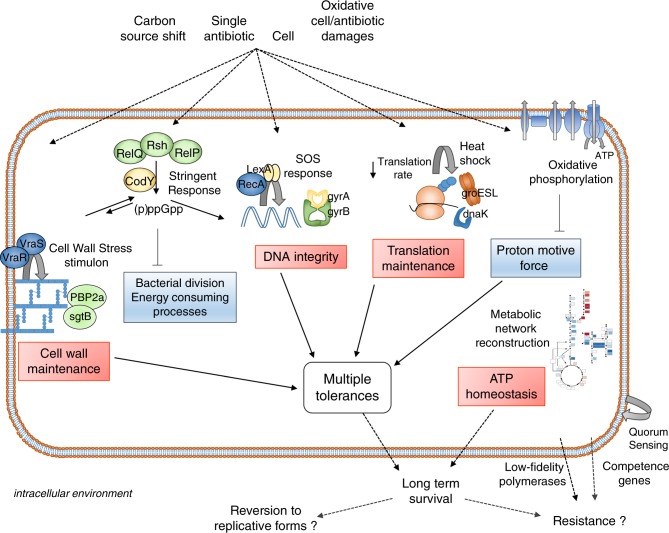
Overview of intracellular persistence regulation of *S. aureus*. In vacuolar nutrient-rich compartments, persisters are metabolically active cells shielding cell wall, DNA and translation products. Under pressure of the environmental factors from the host cell, including a carbon source shift and antibiotic pressure, persisters promote a network of stress or adaptive responses displaying multiple entries. Stringent response does not show signs of activity for prolonged periods but rather contributes partly to initiate the switch to a persister phenotype through (i) post-translational modifications, contributing to an almost immediate blockade of bacterial division, and (ii) transcriptional regulation, silencing energy-consuming processes. Regulation circuits also include the cell wall stress stimulon, the SOS response, and the heat shock response. These active responses, together with a decrease in oxidative phosphorylation and in translation levels, lead to multidrug tolerance upon exposure to a single antibiotic. This stable phenotype allows bacteria to maximize the chances of long-term survival. Finally, depending on the level of stress, this state could either revert to replicative forms, or promote the evolution to resistant forms, through increased probability of mutations and horizontal gene transfer.

Our RNA-sequencing analysis offers a comprehensive overview of the transcriptomic profile of the living population of *S. aureus* persisters. The transcriptomic patterns highlighted in this study led us to propose a model of factors leading to persistence and tolerance (Fig. 6).

A transitory boost of SR contributes to initiate the switch to intracellular persistence in response to a complex set of environmental stresses. Interestingly, no ATP or amino acid limitations occur intracellularly. Noteworthy, neither oxacillin in planktonic cultures nor permissive cells alone could trigger marked *S. aureus* persistence under our conditions, thus indicating that stresses of different nature can collectively initiate persistence when reaching a certain threshold. During infection, bacteria are typically internalized in vacuolar compartments and experience numerous stresses from the host cell and antibiotic pressure. Besides the direct effect of the antibiotic, these stresses include a carbon source transition and a contribution of acidic pH^14^, which may work in concert with cell- and antibiotic-induced oxidative stresses^35,56^.

SR redirects many physiological activities at the expense of those required for growth and proliferation through transcriptional regulation circuits and post-translational modifications that represent a second control point “freezing” the system, conferring extreme plasticity towards external stresses.

We did not find evidence of expression of SR regulators for prolonged periods. Our data rather supports the hypothesis that the mechanism of persistence is not solely dependent on SR but that multiple pathways contribute to persister generation and maintenance. The redundant character of this signaling network might thus result from the continuous and multiple stresses bacteria are facing, rather than a unique regulator.

Our work also questions the concept of tolerance occurring as a passive phenomenon through target inactivity. We showed that intracellular persisters are metabolically active cells, which exhibit a mosaic of adaptive responses that lead to a phenotype of multiple tolerance. These responses seem to redundantly occur, and involve the activation of the CWSS, the SOS and heat shock responses, as well as the induction of several antibiotic targets. This study also demonstrates that exposure to a single drug can trigger a phenotype of multiple tolerance to several antibiotic classes intracellularly. The clinical meaning of these observations remains to be established.

Our model for persistence integrates apparent contradictory observations (e.g., dormancy status or levels of tolerance) often interpreted as physiological diversity^57,58^ (Supplementary Fig. 10). We propose that persistence is highly plastic through essentially redundant regulations, which differentially adapt levels of dormancy as a function of sensed level of stress. In that sense, the proposed heterogeneity should essentially result from differences of degree rather than of nature.

Once intracellular persistence is established, we hypothesize this could constitute a state at the crossroads, either reverting to replicative forms if the level of stress is released, or promoting the evolution to resistant forms if the pressure is maintained^59^. In the latter case, our data indicate that this evolution could be promoted mainly at two distinct levels, through potentiation of higher mutation rates^60^ and extensive horizontal gene transfer^61^, as a consequence of drastic activations of low-fidelity polymerases and competence genes, respectively (Supplementary Fig. 11 and Supplementary Table 3 for summary).

Thus, the present work demonstrates that bacteria surviving to antibiotics intracellularly are persisters which harbor a profoundly reshaped transcriptome. They activate a series of stress responses for long-term survival. Our results therefore highlight persistence as a potential critical trigger for therapeutic failures.

## Methods

### Bacterial strains and cells

Strains used in this study are listed in Supplementary Table 1. *S. aureus* strains were routinely grown at 37 °C in cation-adjusted Mueller-Hinton broth (MHB-CA; Sigma) under shaking at 300 rpm. The fully susceptible strain SH1000 (ref. ^62^) was used to harbor the pALC2084 plasmid, which encodes a reporter *gfp* gene cloned downstream of a *xyl/tetO* promoter and allows a dose-dependent tetracycline induction in vivo and in vitro^63^. GFP production was induced by a sub-inhibitory concentration (125 ng mL^−1^) of tetracycline. Overnight cultures were supplemented with 10 mg L^−1^ chloramphenicol. All experiments were performed on SH1000, unless stated otherwise.

Murine J774A.1 macrophages^64^ (Sandoz Forschung Laboratories) were cultured in RPMI 1640 medium (Thermo Fisher Scientific) supplemented with 10% fetal bovine serum (FBS; Thermo Fisher Scientific) at 37 °C in a 5% CO_2_ atmosphere. When indicated, cells were washed in sterile PBS (filtered on 0.22 µm pore size membrane when used for flow cytometry analysis). Cells were seeded in 12-well plates (Greiner bio-one), in Labtek 2-well Chamber Slide (Nunc) for confocal microscopy, or in 145 mm cell culture dishes (Greiner bio-one) for cell sorting.

Human macrophages were obtained by isolation and differentiation of monocytes from peripheral blood according to the protocol from Menck et al.^65^. Briefly, buffy coats from healthy blood donors were subjected to a double Ficoll and Percoll density gradient centrifugation for isolation of monocytes from peripheral blood. For differentiation to macrophages, monocytes were resuspended in RPMI 1640 with 2% human serum (Biowest), 1% penicillin/streptomycin (Thermo Fisher Scientific) and 2.5 ng mL^−1^ M-CSF (Miltenyl Biotec) and seeded in 12-well plates for 7 days at 37 °C in a 5% CO_2_ atmosphere. Cells were then collected and seeded in 12-well plates in RPMI 1640 with 10% FBS for infection. Human THP-1 monocytes (ATCC TIB-202) were cultured in RPMI 1640 medium with 10% FBS as previously described^66^. Human MG63 osteoblastic cells (LGC Standards) were cultured in Dulbecco’s modified Eagle’s medium (DMEM) with 10% FBS^67^. Human A549 (ATCC CCL-185) and MCF7 (ATCC HTB-22) epithelial cells were cultured in DMEM with 10% FBS and DMEM with 10% FBS and 0.01 mg mL^−1^ insulin (Gibco). Human adult primary keratinocytes were cultured in supplemented EpiLife medium (EpiLife with S7; Thermo Fisher Scientific) in collagen coated plates as previously described^68^.

### Experiments in broth

For Fluorescence dilution experiments, overnight *S. aureus* cultures in MHB-CA supplemented with 125 ng mL^−1^ tetracycline were centrifuged at 5000 *g* for 5 min, washed out in PBS to remove tetracycline, and diluted in fresh medium to reach a starting OD_620 nm_ of 0.05. Cultures were incubated at 37 °C and aliquots were collected over time, centrifuged, washed in PBS, resuspended in filtered PBS and analyzed by flow cytometry or epifluorescence microscopy (Carl Zeiss Axioskop 40) as described below (see also Supplementary Fig. 12 for gating methods). For time-kill curves, *S. aureus* was grown overnight in MHB-CA, diluted in fresh medium to reach a starting OD_620 nm_ of 0.05 and grown to the mid-exponential phase. Cultures were diluted to a starting inoculum of 1 × 10^6^ cfu mL^−1^ and exposed to oxacillin (Sigma), clarithromycin (SMB-Galephar), or moxifloxacin (Sigma) at 50× their respective MIC, for the indicated times. For cfu counting, samples were diluted in PBS before plating on Tryptic Soy agar. Data are expressed as log_10_ cfu mL^−1^ after the incubation period compared to the starting inoculum.

### Infection of macrophages and other cell types

Infection was performed following a protocol adapted from Seral et al.^69^ and Barcia-Macay et al.^66^. *S. aureus* was grown overnight in MHB-CA supplemented with 125 ng mL^−1^ tetracycline. Bacteria were then centrifuged at 5000 *g* for 5 min and resuspended in RPMI 1640 supplemented with 125 ng mL^−1^ tetracycline and 10% human serum to allow opsonization for 30 min at 37 °C. Bacteria were centrifuged, resuspended in fresh RPMI 1640 with 10% FBS and 125 ng mL^−1^ tetracycline, and incubated with cells during 30 min at 37 °C at a multiplicity of infection of 10:1 to allow phagocytosis. The multiplicity of infection (MOI) was adapted to 100:1 for RNA-seq in order to obtain a sufficient amount of bacterial material (see Supplementary Fig. 13 for evaluation of the absence of effect of MOI on the dynamics of replication) and 1:1 for confocal microscopy. Cells were then washed with PBS, and non-phagocytized bacteria were eliminated by a 40 min incubation at 37 °C in RPMI 1640 supplemented with 50 mg L^−1^ gentamicin (Sigma). Gentamicin was eliminated by washing in PBS, after which cells were reincubated at 37 °C in RPMI 1640 with 10% FBS in the presence either of 2× MIC of gentamicin (to prevent extracellular growth; control conditions)^69^, or of oxacillin, clarithromycin, or moxifloxacin at 2 or 50× their respective MIC, for the indicated times. Cells were then washed with PBS, scrapped and lysed with PBS containing 0.1% (w/v) Triton X-100 (Sigma) to release intracellular bacteria. Lysates were centrifuged at 300 *g* for 5 min to discard cellular debris. Bacteria were collected by centrifugation at 5000 *g* for 5 min, washed in PBS, and resuspended in filtered PBS. For cfu counting, samples were diluted in PBS before plating on Tryptic Soy agar. Data are expressed as log_10_ cfu per mg cell protein after the incubation period compared to the post-phagocytosis inoculum. The same protocol was applied for infection of primary human macrophages and THP-1 monocytes, except that THP-1 cells were growing in suspension, so that washing procedures involved cell pelleting by centrifugation (7 min, 300 *g*). For other cell types (A549, MCF7, MG63 and primary keratinocytes), the same protocol was applied as for macrophages, except that bacteria were incubated with cells during 2 h at a multiplicity of infection of 50:1. This allowed to reach typical inocula of 2 × 10^6^ bacteria mg^−1^ of cell protein for macrophages and monocytes, 0.4 × 10^6^ bacteria per mg of cell protein for A549, MCF7 and MG63, and 0.2 × 10^6^ bacteria per mg of cell protein for keratinocytes. All experiments were performed on SH1000 induced for GFP expression, with the exception of experiments on the rate of GFP synthesis (see hereafter).

### Rate of GFP synthesis in intracellular persisters

Macrophages were infected by non-induced bacteria following the same procedure and incubated with or without 50× MIC oxacillin for 24 h, after which induction were performed with 125 ng mL^−1^ tetracycline for the indicated periods.

### Confocal microscopy

Infected macrophages seeded in Labtek chambers were incubated with gentamicin or moxifloxacin in RPMI 1640 without phenol red. Prior to microscopy, chambers were removed and slides covered with cover glasses. Infected cells were observed with a Cell Observer SD (Carl Zeiss) and analyzed with Zen v1.1.2.0 software (Carl Zeiss).

### Flow cytometry

*S. aureus* isolated from broth cultures or from macrophages were resuspended in filtered PBS, stained with 10 µg mL^−1^ propidium iodide, and analyzed using a FACSVerse cytometer (BD Biosciences) for GFP signal intensities (FITC channel, medium flow rate). Forward-scatter width (FCS-W) versus forward-scatter area (FSC-A), and side-scatter width (SSC-W) versus side-scatter area (SSC-A) were used to gate out damaged or multiplet cells. Of those, propidium iodide-positive bacteria were gated out. Data were analyzed with FlowJo 10.5.2 software (TreeStar Inc.). The level of replication of the population (*F*, fold replication) is calculated by the ratio Me_0_/Me_t_ with Me being the median GFP intensity of the bacterial population at a given time. The number of generations, *N*, is deduced from *F* = 2^*N*^ (ref. ^19^).

### Samples preparation for RNA-seq

For differential expression analysis, bacterial reference samples (hereafter named “control samples” in RNA-seq experiments) were collected from an overnight culture (MHB-CA supplemented with 125 ng mL^−1^ tetracycline) by centrifugation at 5000 *g* for 5 min, resuspended in RPMI 1640 and incubated for 30 min at 37 °C. This bacterial suspension was then mixed with a cell lysate obtained from non-infected macrophages incubated in RPMI 1640 with 10% FBS for 24 h, in order to mimic the matrix effect of macrophages in the persister condition. The relative amount of bacteria and cells was adjusted to that obtained in 24 h-infected cells. Intracellular persisters were collected from macrophages as described for flow cytometry. Both control and persisters conditions were proceeded for the same sorting procedure. Bacteria were immediately proceeded for sorting on the basis of their propidium iodide profile and GFP expression level using the gating methods described above, in a FACSAria III cytometer operated by the BD FACSDiva 8.0.1 software (BD Biosciences), under continuous cooling to 4 °C (including the input tube holder and the collection tube) at high flow rate. Samples were then immediately processed for RNA extraction.

### RNA extraction

*S. aureus* recovered from infected macrophages or from control samples were centrifuged at 5000 *g* for 5 min and lysed using the following procedure: pellets were resuspended in Tris-EDTA buffer containing freshly prepared 13 mg mL^−1^ lysozyme (Sigma) and 130 µg mL^−1^ lysostaphin (Sigma) for 30 min at room temperature. Resulting suspensions were processed for total RNA extraction with RNA extraction InviTrap Spin Universal RNA Mini Kit (Stratec) following the manufacturer’s instructions. Traces of contaminating genomic DNA were removed from samples by treatment with TURBO DNase (Ambion) for 30 min at 37 °C according to the manufacturer’s instructions. RNA purity was checked using a NanoDrop spectrophotometer (Thermo Fisher Scientific).

### RNA sequencing

Total RNA from three independent replicates were checked on the Bioanalyser system (Agilent) for its quality and integrity. Ribosomal RNA depletion was performed using the Bacteria RiboZero kit (Illumina). From rRNA-depleted RNA, directional libraries were prepared using the TruSeq Stranded mRNA Sample preparation kit following the manufacturer’s instructions (Illumina). Libraries were checked for quality on Bioanalyser DNA chips Bioanalyser (Agilent). Quantification was performed with the fluorescent-based quantitation Qubit dsDNA HS Assay Kit (Thermo Fisher Scientific). Sequencing was performed as an SRM run (SR: Single Read, PE: Paired-end Reads, M: multiplexed samples) for 65 bp sequences on HiSeq 2500 Illumina sequencer (65 cycles). The multiplexing level was 6 samples per lane. Bioinformatics analysis were performed using the RNA-seq pipeline from Sequana^70^. Reads were cleaned of adapter sequences and low-quality sequences using cutadapt version 1.11 (ref. ^71^). Only sequences at least 25 nt length were considered for further analysis. Bowtie version 0.12.7 (ref. ^72^) with default parameters, was used for alignment on the reference genome (CP000253.1, NCBI). Genes were counted using featureCounts version 1.4.6-p3 (ref. ^73^) from Subreads package (parameters: -t gene -g ID -s 1). Count data were analyzed using R version 3.4.1 (ref. ^74^) and the Bioconductor package DESeq2 version 1.16 (ref. ^75^). The normalization and dispersion estimation were performed with DESeq2 using the default parameters and statistical tests for differential expression were performed applying the independent filtering algorithm. A generalized linear model was set in order to test for the differential expression between the intracellular persisters and control conditions. Raw *P* values were adjusted for multiple testing according to the Benjamini and Hochberg (BH) procedure^76^ and genes with an adjusted *p* value lower than 0.05 were considered differentially expressed. For over-representation analysis, *S. aureus* KEGG gene-sets were downloaded thanks to the EnrichmentBrowser R package version 2.14.3 (organism code sao). All the 106 KEGG sets were then tested for the over-representation in differentially expressed genes using the Fisher statistical test. Only gene-sets with a FDR lower than 0.05 were considered significantly enriched.

### Quantitative real-time PCR

Total bacterial RNA from infected macrophages or from control samples at different time points was isolated as described for RNA-seq analyses. RNA was reverse transcribed using transcription first strand cDNA synthesis kit (Roche Applied Science) according to the manufacturer’s instructions. Amplification reactions were performed with Sybr green IQ Supermix (Bio-Rad Laboratories) using an iCycler iQ single-color real-time PCR detection system (Bio-Rad Laboratories). Fold changes in expression versus control condition were determined using the 2^(−ΔΔCt)^ method^77^ with *gmk* as a housekeeping gene. Primers sequences are listed in Supplementary Table 4.

### ATP measurements

Bacteria were released from macrophages as described above, washed in 50 mM Tris-HCl, centrifuged at 5000 *g* for 5 min and processed for lysis following the same procedure as for RNA extraction. Control samples (as described for RNA-seq experiments) were used for comparison purposes. Bacterial lysates were incubated 2 min at 100 °C, centrifuged at 9600 *g* for 2 min and assayed for ATP measurements, using the ATP determination kit (Thermo Fisher Scientific) according to the manufacturer’s instructions. Bioluminescence was measured using a SpectraMax M3 548 Microplate Reader (Molecular Devices).

### Ethics statement

Experiments on blood material were performed in strict accordance with governmental and European legislation relative to blood, cell and tissues-related activities, and were approved by the ethical committee *Comité d’Ethique Hospitalo-Facultaire Saint-Luc* (CEHF Saint-Luc; permit no. B403201730810). Human blood was collected in *Croix-Rouge de Belgique* centers, from healthy volunteers who gave written informed consent, in accordance with procedures of *Service Francophone du Sang de la Croix–Rouge de Belgique*.

### Statistical analysis and curve fitting

For RNA-seq analyses, differential expressions of transcripts were based on adjusted *P*-values with a threshold of statistical significance set to 0.05. Genes descriptions were indicated as described in GenBank database^23^ and classified according to KEGG orthology^78^ and KEGG pathway^79^ databases (organism code CP000253.1 and sao respectively). Curve fitting and statistical analyses were performed with GraphPad Prism versions 4.03 or 8.3.1, GraphPad InStat v3.10 (GraphPad Software), and JMP Pro version 13.1.0. Statistical differences were determined using unpaired two-tailed Student’s *t*-tests or one-way ANOVA with Dunnett’s post-tests for multiple comparisons. *P-*values strictly inferior to 0.05 were used to show statistical significance and are indicated in Figures. Non-statistically significant differences are indicated as “ns”.

### Reporting summary

Further information on research design is available in the Nature Research Reporting Summary linked to this article.

## Supplementary information

Supplementary Information

Reporting Summary

## Data Availability

The source data underlying Figs. 1a, b, e, h, i, l, 3a, b, d, 4a, d, 5a and f are provided as a Source Data file. RNA-seq data reported in this study have been deposited at Gene Expression Omnibus under accession number GSE139659 [https://www.ncbi.nlm.nih.gov/geo/query/acc.cgi?acc=GSE139659]. All other relevant data are available from the corresponding author on request.

## Source Data

Source Data
