# Supplementary material for: Intracellular Staphylococcus aureus persisters upon antibiotic exposure
Source: Nat Commun. 2020 May 4;11:2200. doi: 10.1038/s41467-020-15966-7 (PMC7198484; doi:10.1038/s41467-020-15966-7)
Supplement: Supplementary file 1 — Supplementary Information [file 41467_2020_15966_MOESM1_ESM.pdf]

Supplementary information

Intracellular *Staphylococcus aureus* persisters upon antibiotic exposure

Peyrusson et al.

Supplementary Figures

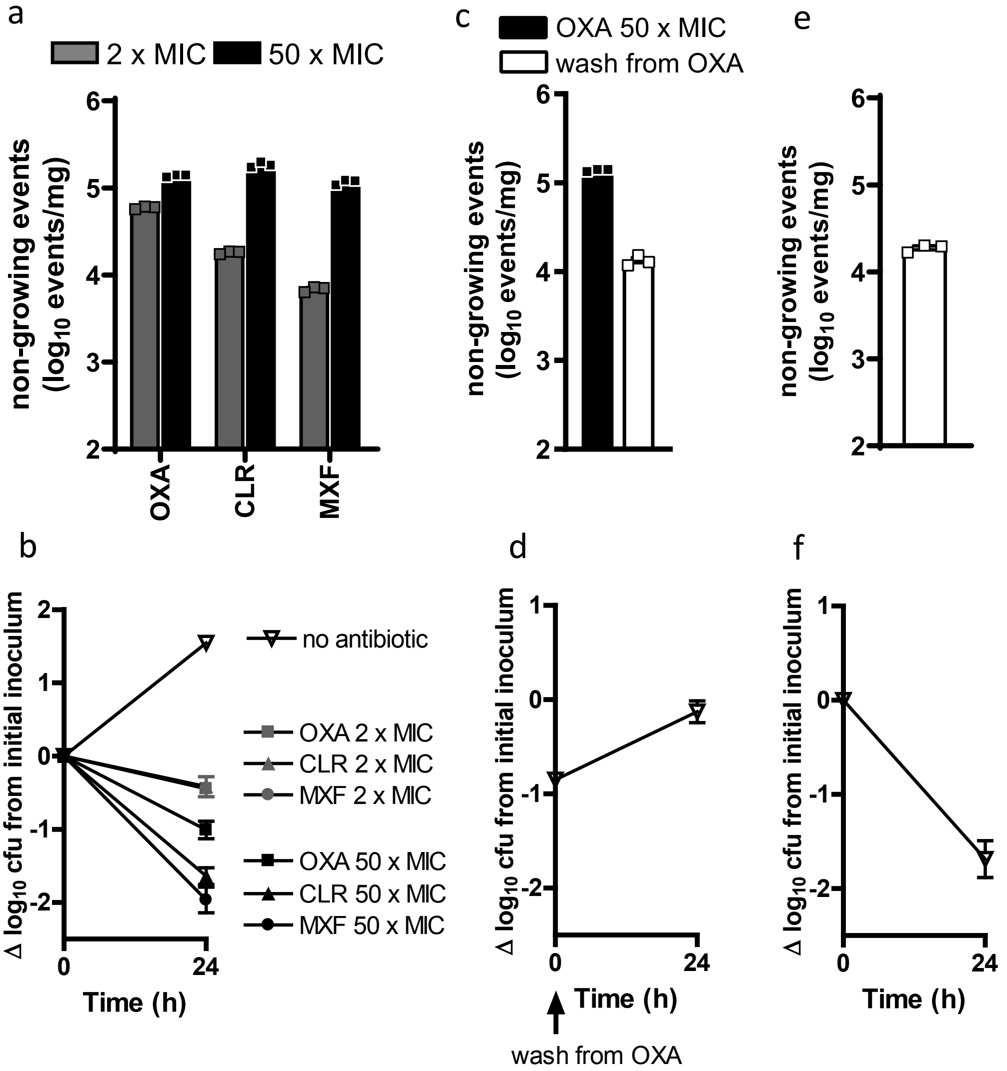

Supplementary Figure 1. Quantification of non-growing events and cfu counts in macrophages. a, Number of non-growing events in flow cytometric profiles of bacteria recovered from J774 macrophages exposed to 2 x or 50 x MIC of each antibiotic for 24 h (corresponding to experiments shown in Figure 1g), and **b,** corresponding cfus. For non-growing events, populations were gated so that over 90% of the induced initial inoculum was above the cutoff (corresponding to 2.10^3 of GFP fluorescence), and normalized to protein content. For cfus, data are expressed as log_{10} cfu reduction from the original inoculum. **c**, Number of non-growing events in flow cytometric profiles of bacteria recovered from J774 macrophages exposed to 50 x MIC oxacillin for 24 h, then washed from oxacillin and reincubated in the absence of antibiotic for an additional period of 24 h (corresponding to experiments shown in Figure 1j), and **d,** corresponding cfus. **e**, Number of non-growing events in flow cytometric profiles of bacteria recovered from untreated human macrophages for 24 h [2 x MIC gentamicin] (corresponding to experiments shown in Figure 1k), and **f,** corresponding cfus. **a-f**,Data are means ± SEM of three independent experiments. Oxacillin [OXA], clarithromycin [CLR], moxifloxacin [MXF].

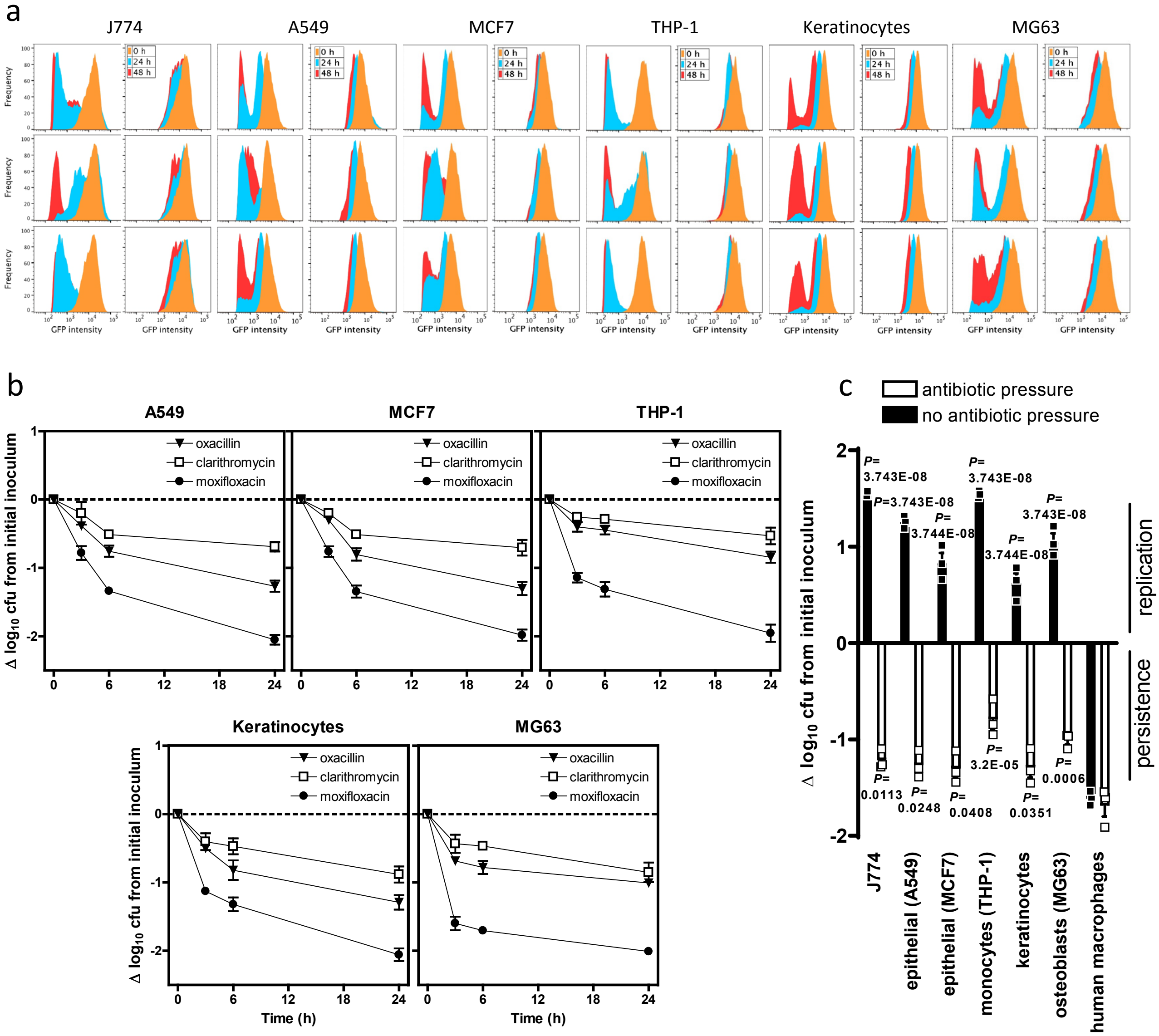

Supplementary Figure 2. Permissive cells as a model for intracellular persistence of *S. aureus*. a, Flow cytometric profiles of *S. aureus* recovered from human epithelial cells (A549 and MCF7), monocytes (THP-1), osteoblasts (MG63) and primary keratinocytes, exposed to 2 x (left) or 50 x MIC (right) of each antibiotic for the indicated periods (representatives results of three independent experiments). **b,** Time-kill curves of intracellular *S. aureus* in the same cell types. Infected cells were exposed to 50 x MIC of oxacillin, clarithromycin, or moxifloxacin for the indicated periods. Data (expressed as $\log_{10}$ cfu reduction from the original inoculum) are means ± SEM of three independent experiments. **c,** Intracellular *S. aureus* recovered from infected cells incubated for 24 h with or without 50 x MIC oxacillin (2 x MIC gentamicin). Data are means ± SEM of three independent experiments. Statistical significance between cells and human macrophages was determined by one-way ANOVA with Dunnett's post-test.

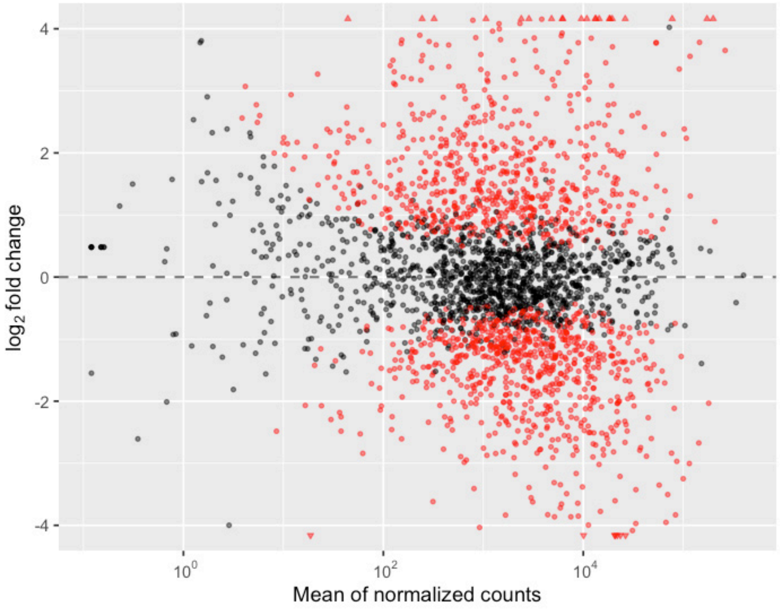

Supplementary Figure 3. MA-plot of complete RNA-seq dataset. The graph displays the $\log_2$ Fold Change expression as a function of mean counts across all samples. Red dots represent significantly differentially expressed features, based on adjusted *P* value.

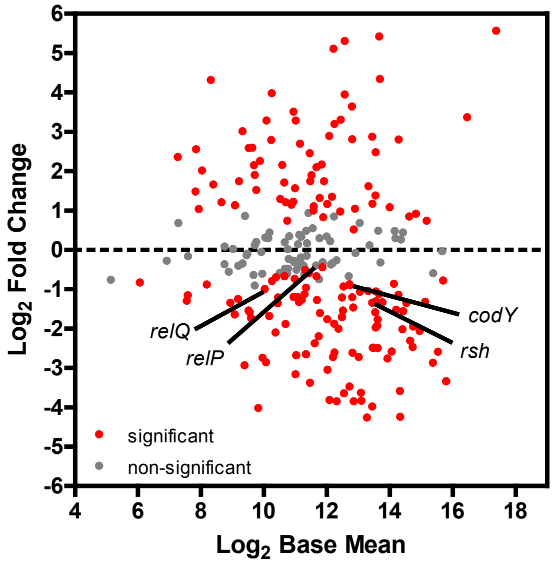

Supplementary Figure 4. MA-plot of genes related to the stringent response stimulon [1], extended to *relP*, *relQ* and *codY*. The graph displays the $\log_2$ Fold Change expression as a function of $\log_2$ Base Mean (mean expression signal across all samples). Typical members of the stimulon are pointed. The dotted lines indicate the basal expression level in control samples. Statistical significance is based on adjusted *P* value.

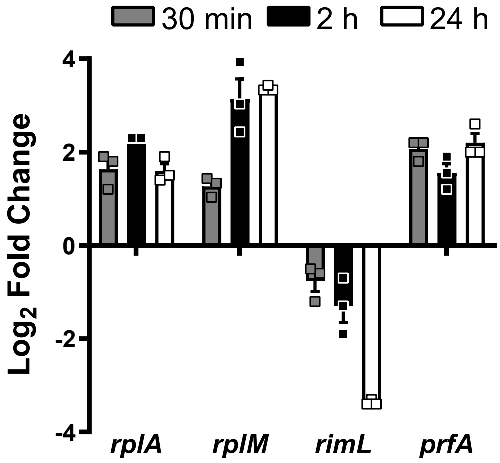

Supplementary Figure 5. Quantitative real-time PCR of transcripts related to translation. Quantitative real-time PCR of transcripts related to translation in intracellular persisters exposed to 50 x MIC oxacillin for the indicated times. Data, expressed in fold change *vs* control samples (extracellular bacteria mixed with J774 cells lysate), are means ± SEM of three independent experiments. Ribosomal proteins: *rplA*, 50S ribosomal protein L1; *rplM*, 50S ribosomal protein L13. Processing protein: *rimL*, 50S ribosomal protein L7 serine acetyltransferase. Translation factor: *prfA*, peptide chain release factor 1.

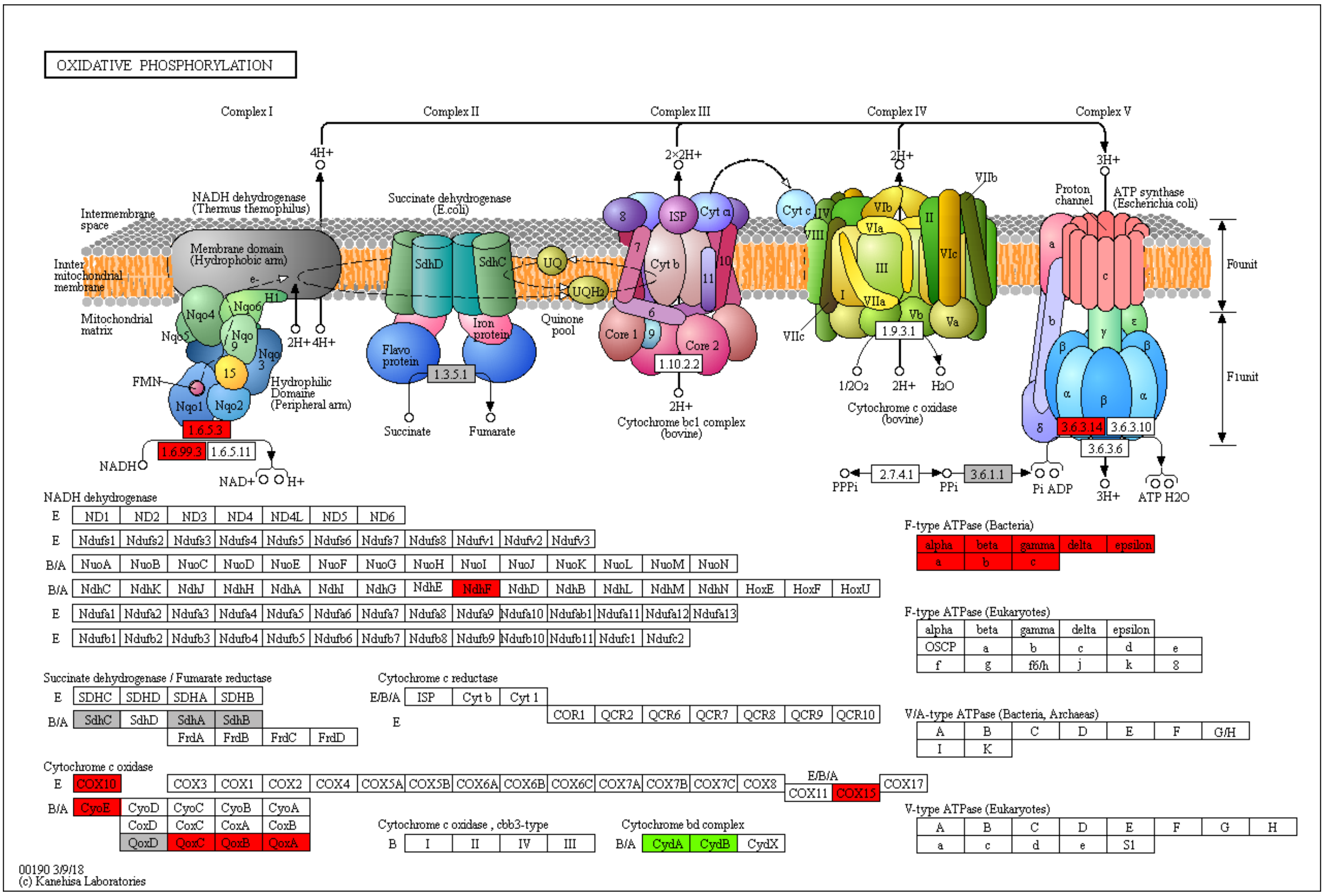

Supplementary Figure 6. KEGG Pathview representation of genes related to oxidative phosphorylation. Fold Changes expressions levels of up-, downregulated and non-statistically modified genes are shown in green, red and grey, respectively.

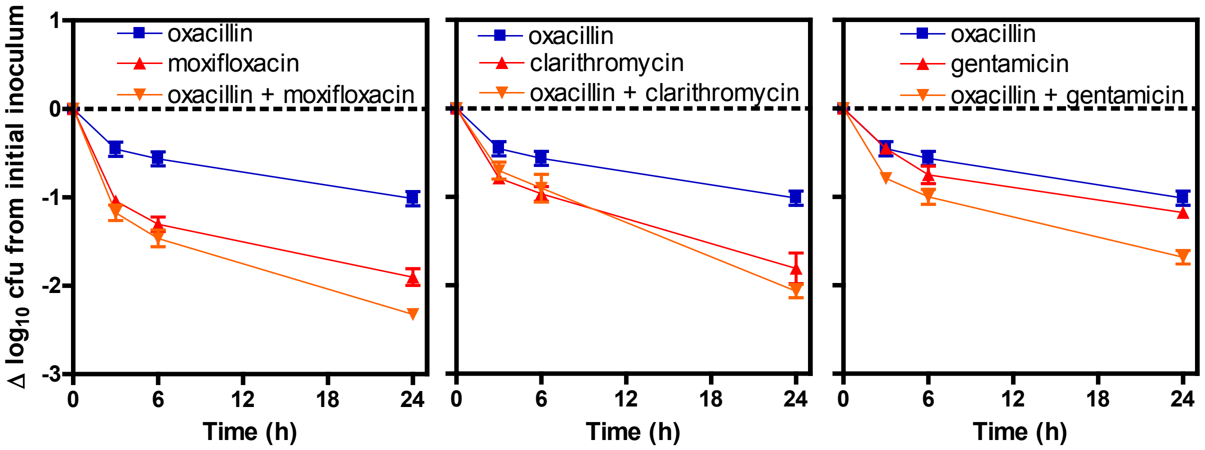

Supplementary Figure 7. Time-kill curves of intracellular *S. aureus*. Infected J774 macrophages were exposed to 50 x MIC of oxacillin, clarithromycin, or moxifloxacin alone or in combination for the indicated periods. Data (expressed as $\log_{10}$ cfu reduction from the original inoculum) are means ± SEM of three independent experiments.

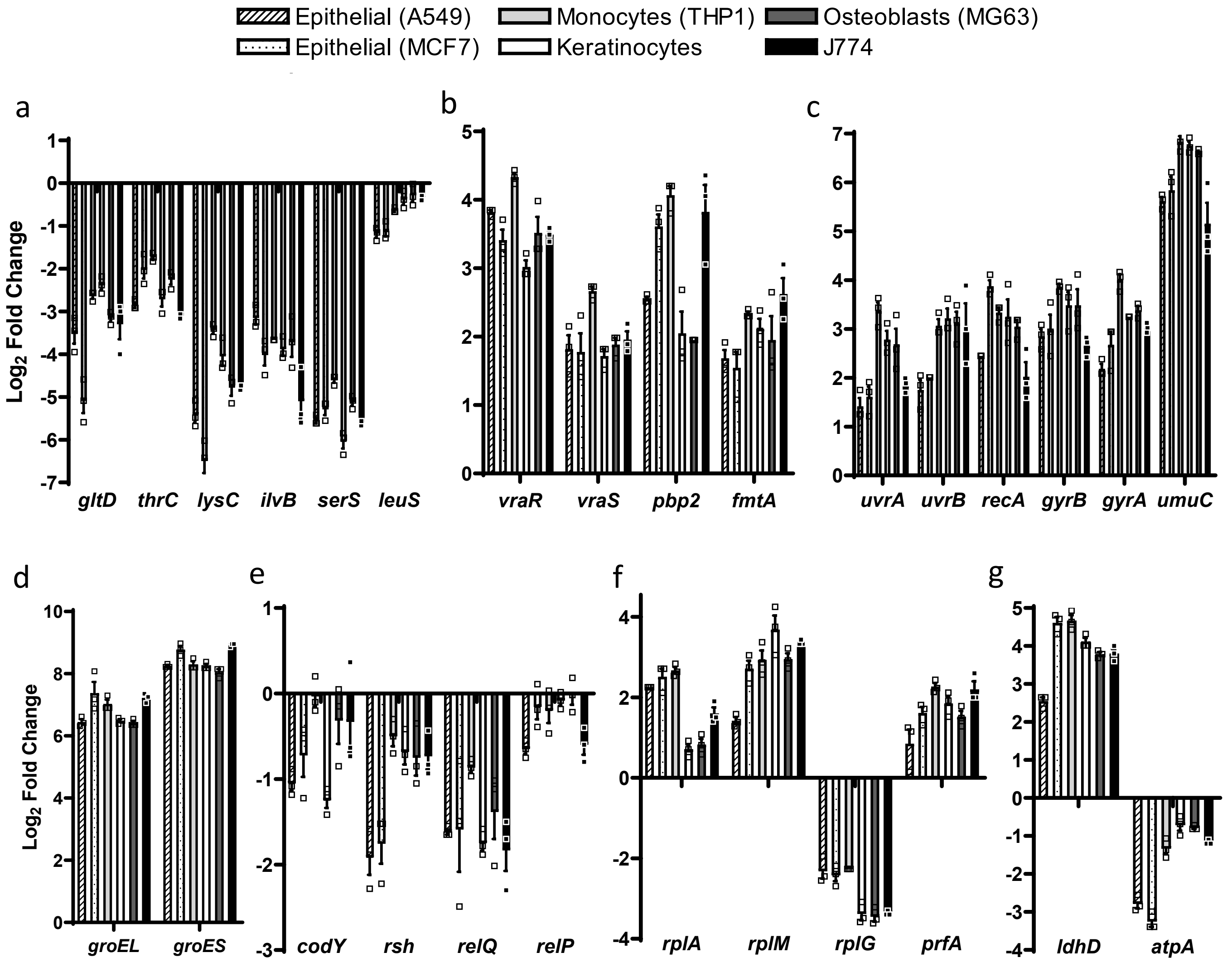

Supplementary Figure 8. Quantitative real-time PCR of transcripts related to amino acid metabolism (**a**), Cell Wall Stress Stimulon (**b**), SOS response (**c**), heat shock stimulon (**d**), stringent response (**e**), translation process (**f**) and energy metabolism (**g**), in intracellular persisters recovered from infected cells, exposed to 50 x MIC oxacillin for 24h. Data, expressed in fold change vs control samples (extracellular bacteria mixed with cells lysates), are means ± SEM of three independent experiments.

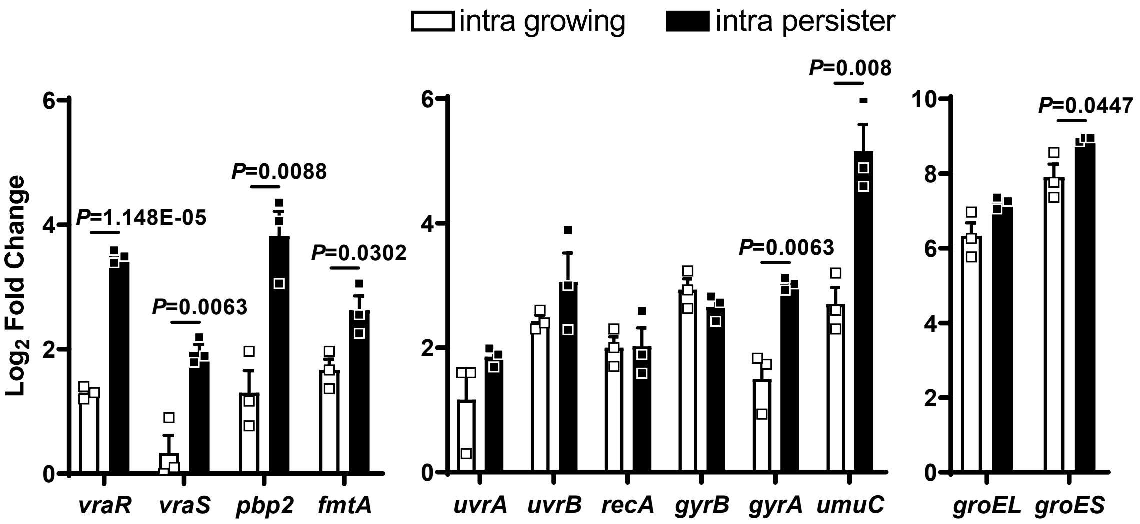

Supplementary Figure 9. **Quantitative real-time PCR of transcripts related to Cell Wall Stress Stimulon, SOS response and heat shock stimulon**, from left to right, in intracellular bacteria with (intra persister) or without (intra growing) 50 x MIC oxacillin, for 24h of infection. Data, expressed in fold change *vs* control samples (extracellular bacteria mixed with J774 cells lysate), are means ± SEM of three independent experiments. Statistical significance was determined by two-tailed Student's t-test.

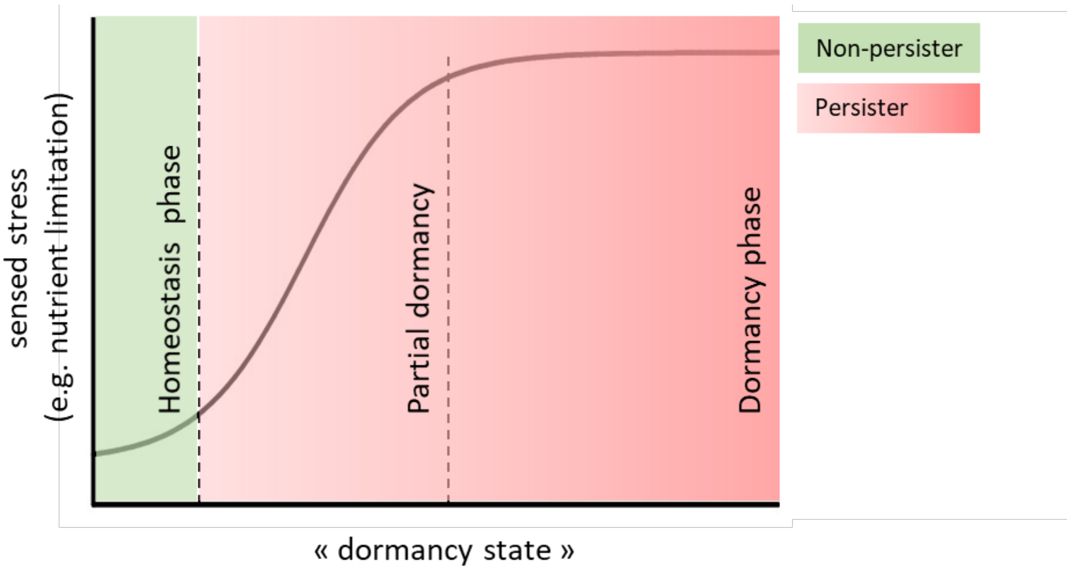

Supplementary Figure 10. Schematic view illustrating the plasticity of persistence. Persistence is regulated through essentially redundant but highly plastic systems, which adapt their magnitude as a function of the sensed level of stress. In this model, persistence can be thought as a continuum in which regulations mechanisms essentially differ in terms of magnitude, leading to different levels of dormancy or tolerance, generally considered as heterogeneity. This notably tends to reconcile different situations encountered in culture media and intracellularly. Various stress factors work in concert to initiate the switch to persistence when a "threshold" is reached. Stress could be then differentially sensed trough balanced responses, which determines the level of dormancy as well as tolerance. Under this limit, bacteria maintain metabolic homeostasis and growth. Once the threshold is reached, bacteria enter in persister state with only partial dormancy, growth inhibition and a sustained ATP charge (e.g. intracellular persisters, or persisters induced following a nutrient shift). In critically lowered nutrient sources, as encountered in starvation models of stationary phases, metabolic adaptation is not sufficient to cope with carbon sources deprivation or other stresses. Bacteria then reach a stasis phase, accompanied by a drop in ATP content. Similar observations are made for tolerance levels which are the reflect of the magnitude of stringent response [2] or other stress responses (e.g. cell wall stress stimulon [3]).

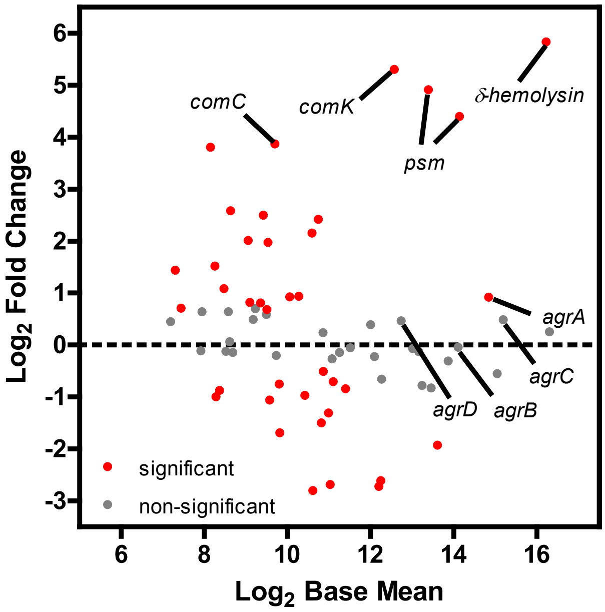

Supplementary Figure 11. MA-plot of genes related to quorum sensing [4]. The graph displays the $\log_2$ Fold Change expression as a function of $\log_2$ Base Mean (mean expression signal across all samples). Typical members of the function are pointed. The dotted lines indicate the basal expression level in control samples. Statistical significance is based on adjusted *P* value.

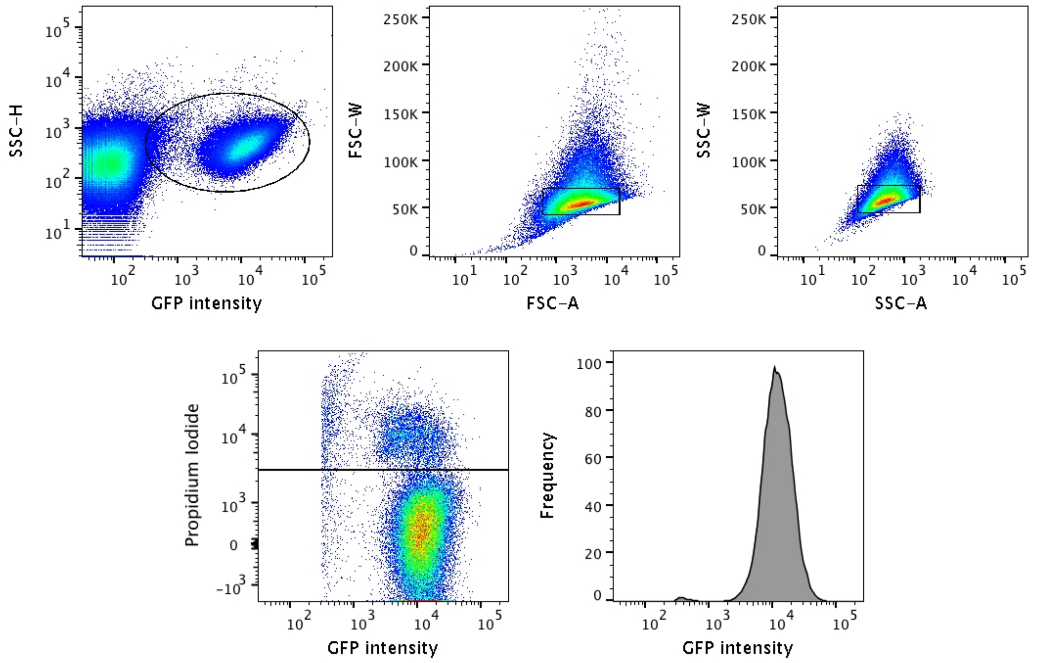

Supplementary Figure 12. Gating methods for fluorescence dilution analyses. Bacteria recovered from macrophages were analyzed by gating forward-scatter width (FCS-W) versus forward-scatter area (FSC-A), and side-scatter width (SSC-W) versus side-scatter area (SSC-A), to gate out damaged or multiplet cells. Propidium iodide-negative events were used for flow cytometric profiles of GFP intensity to measure bacterial replication. The same gating methods were used for all experiments (as shown or interpreted in Figure 1c, e, g, j, k; Supplementary Figure 1a, c, e and Supplementary Figure 2a) and for sorting strategy (as presented in Figure 2a).

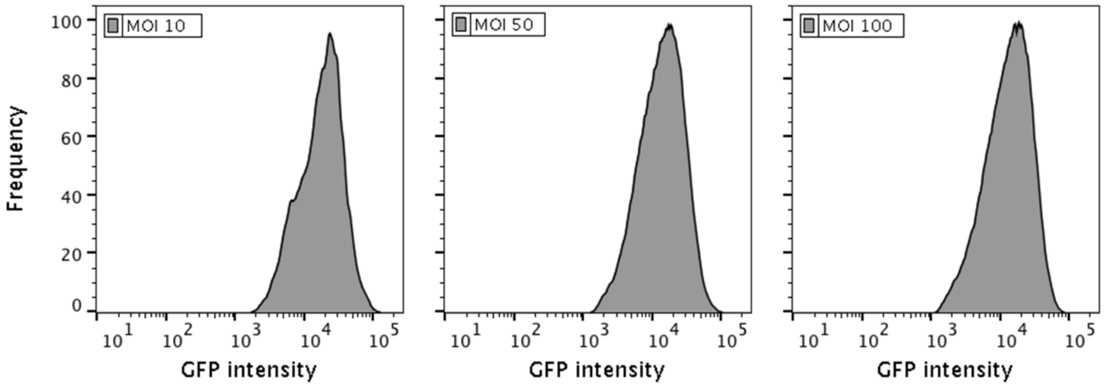

Supplementary Figure 13. Effect of multiplicity of infection (MOI) on dynamics of intracellular persisters. *S. aureus* persisters from infected macrophages at different MOI, exposed for 24 h to 50 x MIC oxacillin were collected and analyzed for their replication status.

Supplementary Tables

Supplementary Table 1. Strains used in this study and MICs of antibiotics.

**Strain**		**Description**			**Reference**
SH1000 pALC2084		SH1000 (*rsbU* restored NCTC 8325 [5]) with *gfp* under tetracycline inducible promoter, CAM^r (pALC2084 [6])			this study
HG001		*rsbU* restored NCTC 8325			[7]
HG001-86		HG001 *rshSyn* mutant (Δ942-950nt)			[8]
HG001-21		HG001 *codY* mutant			[7]
HG001-86-21		HG001 *rshSyn* (Δ942-950nt)/*codY* double mutant			[8]
		**MIC (mg L^{-1})**			
**Strain**	oxacillin	clarithromycin	moxifloxacin	gentamicin	
SH1000 pALC2084	0.5	0.5	0.03	0.5	
HG001	0.25	0.5	0.03	0.25	
HG001-86	0.25	0.5	0.03	0.25	
HG001-21	0.25	0.5	0.03	0.5	
HG001-86-21	0.25	0.5	0.03	0.5	

Supplementary Table 2. Overview of main stress responses and postulated consequences on the observed tolerance phenotype.

**Active stimulon/function**	**Gene(s)**	**Postulated effect on phenotype**
Cell Wall Stress Stimulon	[9], including penicillin binding protein *pbp2* and *fmtA* (Figure 5b)	tolerance to β-lactams [3]
SOS response	[1], including fluoroquinolones targets *gyrA-B* and *parC-E* (Figure 5c)	tolerance to fluoroquinolones [10]
Heat shock response	[1] (Figure 5e)	tolerance to β-lactams [11, 12], aminoglycosides [13] and possibly macrolides (Pth-mediated peptidyl-tRNA processing) [14]
**Repressed function**		
SOS response	[1] *ftsz* (cell division protein)	tolerance to β-lactams [15]
Energy metabolism	electron transport chain (Supplementary Figure 6)	tolerance to aminoglycosides (decreased proton motive force) [16]
Translation	[4] (Figure 3c)	tolerance to aminoglycosides/macrolides, indirect tolerance to β-lactams and fluoroquinolones (decreased translation rate) [7, 17-19]

Supplementary Table 3. Overview of main genes upon persistence possibly implicated in evolution to resistance phenotype.

Activated gene(s)	FC expression	Effect on phenotype
Low fidelity polymerase V *umuC* (Figure 5c)	20.1	SR- and SOS-mediated adaptive mutagenesis resistance to fluoroquinolones [20]
Low fidelity polymerase IV *dinB* (Figure 5c)	2.2	SOS-mediated adaptive mutagenesis resistance to fluoroquinolones [20]
Competence genes (*comK*, *comC*) (Supplementary Figure 11)	39.1; 14.5	SR-mediated (*comK*) Competence, increase horizontal gene transfer frequency [21]
vraR-*vraS* two-component system	4.5; 5.3	CWSS control resistance to cell wall-acting agents [3]
methicillin resistance protein *fmtA* (Figure 5b)	9.1	SR- and CWSS-mediated methicillin resistance [22]
methicillin resistance protein *fmtB*	3.3	methicillin resistance [23]
methicillin resistance determinant protein *fmhA*	6.8	methicillin resistance [24]
MATE transporter *mepA*	11.3	multidrug resistance [25]
MFS transporters (*SAOUHSC_02531* ; *sdrM* ; *lmrS*)	21.0 ; 3.2 ; 1.6	multidrug resistance [25]

Supplementary Table 4. Primers for quantitative real-time PCR.

Description	Template [26]	Sequence (forward)	Sequence (reverse)
codY	SH1000	GGTGGAGGGGAAAGATTAGG	GCGCGCTTCTTTTTCTACTT
rsh	SH1000	CCCCAGCGAGTGATGTTATT	AATTTTGCCATTCACCTTGG
relQ	SH1000	TATGGAGCGACGTGTGAAAG	GAATTCCGGCAATATCCAGT
relP	SH1000	AAGCAAGCGGTTGATGAGTT	CGATTGGTTTAACACGACCAG
gltD	SH1000	AAAGTGGCAATCGTTGGAAG	AACCGCCTGATTCTCTAGCA
thrC	SH1000	AATGTTTGGCTTCCAAGCTG	CGCCTTATCCCAACTAGCAG
thrA	SH1000	TGTATGCCACTGACCCAAGA	GCACCTAAAGCGCTCATTTC
ilvB	SH1000	ACAGACGTGGGACAACATCA	GCACCAATTGACGAAGGAAT
serS	SH1000	GTGAAGCAGGATCAGCAGGT	TTGCTTCTGCGTTTGTTGTC
leuS	SH1000	GTTGGACATCCTGAGGGCTA	CGTTGCCAGTGTCTAAAGCA
rplA	SH1000	CACCAGACATGATGGGTGAA	ACCAGCTTTTTCAGCACGAT
rplM	SH1000	GATGCTGAAGGCCAAACATT	CCAGTATCAACGTGTGGTGTG
rimL	SH1000	CACAAGCAGTTGAGGCATTG	AATCCCAGCCTTTCAGGAAT
prfA	SH1000	GCGATTTTTGCTGGTGATTT	CCACCATGGTCACTTTCAGA
gmk	SH1000	AAGGTGCAAAGCAAGTTAGAA	CTTTACGCGCTTCGTTAATAC

Supplementary References

1. Anderson, K.L., et al., *Characterization of the Staphylococcus aureus heat shock, cold shock, stringent, and SOS responses and their effects on log-phase mRNA turnover.* J Bacteriol. **188**(19): p. 6739-56 2006
2. Amato, S.M. and M.P. Brynildsen, *Persister Heterogeneity Arising from a Single Metabolic Stress.* Curr Biol. **25**(16): p. 2090-8 2015
3. Dengler, V., et al., *Induction kinetics of the Staphylococcus aureus cell wall stress stimulon in response to different cell wall active antibiotics.* BMC Microbiol. **11**: p. 16 2011
4. *KEGG Orthology - Staphylococcus aureus subsp. aureus NCTC8325*. Available from: https://www.genome.jp/kegg-bin/get_htext?sao00001.keg.
5. Horsburgh, M.J., et al., *sigmaB modulates virulence determinant expression and stress resistance: characterization of a functional rsbU strain derived from Staphylococcus aureus 8325-4.* J Bacteriol. **184**(19): p. 5457-67 2002
6. Bateman, B.T., et al., *Evaluation of a tetracycline-inducible promoter in Staphylococcus aureus in vitro and in vivo and its application in demonstrating the role of sigB in microcolony formation.* Infect Immun. **69**(12): p. 7851-7 2001
7. Pohl, K., et al., *CodY in Staphylococcus aureus: a regulatory link between metabolism and virulence gene expression.* J Bacteriol. **191**(9): p. 2953-63 2009
8. Geiger, T., et al., *Role of the (p)ppGpp synthase RSH, a RelA/SpoT homolog, in stringent response and virulence of Staphylococcus aureus.* Infect Immun. **78**(5): p. 1873-83 2010
9. Utaida, S., et al., *Genome-wide transcriptional profiling of the response of Staphylococcus aureus to cell-wall-active antibiotics reveals a cell-wall-stress stimulon.* Microbiology. **149**(Pt 10): p. 2719-32 2003
10. Dorr, T., K. Lewis, and M. Vulic, *SOS response induces persistence to fluoroquinolones in Escherichia coli.* PLoS Genet. **5**(12): p. e1000760 2009
11. Singh, V.K., et al., *Role for dnaK locus in tolerance of multiple stresses in Staphylococcus aureus.* Microbiology. **153**(Pt 9): p. 3162-73 2007
12. Murakami, K., et al., *Role for rpoS gene of Pseudomonas aeruginosa in antibiotic tolerance.* FEMS Microbiol Lett. **242**(1): p. 161-7 2005
13. Cardoso, K., et al., *DnaK and GroEL are induced in response to antibiotic and heat shock in Acinetobacter baumannii.* J Med Microbiol. **59**(Pt 9): p. 1061-8 2010
14. Das, G. and U. Varshney, *Peptidyl-tRNA hydrolase and its critical role in protein biosynthesis.* Microbiology. **152**(Pt 8): p. 2191-5 2006
15. Miller, C., et al., *SOS response induction by beta-lactams and bacterial defense against antibiotic lethality.* Science. **305**(5690): p. 1629-31 2004
16. Kohanski, M.A., D.J. Dwyer, and J.J. Collins, *How antibiotics kill bacteria: from targets to networks.* Nat Rev Microbiol. **8**(6): p. 423-35 2010
17. Davis, B.D., *Mechanism of bactericidal action of aminoglycosides.* Microbiol Rev. **51**(3): p. 341-50 1987
18. Keren, I., et al., *Specialized persister cells and the mechanism of multidrug tolerance in Escherichia coli.* J Bacteriol. **186**(24): p. 8172-80 2004
19. Wilmaerts, D., et al., *General Mechanisms Leading to Persister Formation and Awakening.* Trends Genet. **35**(6): p. 401-411 2019
20. Mesak, L.R., V. Miao, and J. Davies, *Effects of subinhibitory concentrations of antibiotics on SOS and DNA repair gene expression in Staphylococcus aureus.* Antimicrob Agents Chemother. **52**(9): p. 3394-7 2008
21. Fagerlund, A., P.E. Granum, and L.S. Havarstein, *Staphylococcus aureus competence genes: mapping of the SigH, ComK1 and ComK2 regulons by transcriptome sequencing.* Mol Microbiol. **94**(3): p. 557-79 2014

22. Komatsuzawa, H., et al., *Characterization of fmtA, a gene that modulates the expression of methicillin resistance in Staphylococcus aureus.* Antimicrob Agents Chemother. **43**(9): p. 2121-5 1999
23. Komatsuzawa, H., et al., *Tn551-mediated insertional inactivation of the fmtB gene encoding a cell wall-associated protein abolishes methicillin resistance in Staphylococcus aureus.* J Antimicrob Chemother. **45**(4): p. 421-31 2000
24. Tschierske, M., et al., *Identification of three additional femAB-like open reading frames in Staphylococcus aureus.* FEMS Microbiol Lett. **171**(2): p. 97-102 1999
25. Yilmaz, C. and G. Ozcengiz, *Antibiotics: Pharmacokinetics, toxicity, resistance and multidrug efflux pumps.* Biochem Pharmacol. **133**: p. 43-62 2017
26. *GenBank - Staphylococcus aureus subsp. aureus NCTC 8325, complete genome*. Available from: https://www.ncbi.nlm.nih.gov/nuccore/CP000253.1.
