# Supplementary material for: Intracellular Staphylococcus aureus persisters upon antibiotic exposure
Source: Nat Commun. 2020 May 4;11:2200. doi: 10.1038/s41467-020-15966-7 (PMC7198484; doi:10.1038/s41467-020-15966-7)
Supplement: Supplementary file 2 — Reporting Summary [file 41467_2020_15966_MOESM2_ESM.pdf]

nature research

Corresponding author(s): Françoise Van Bambeke

Last updated by author(s): Mar 26, 2020

Reporting Summary

Nature Research wishes to improve the reproducibility of the work that we publish. This form provides structure for consistency and transparency in reporting. For further information on Nature Research policies, see Authors & Referees and the Editorial Policy Checklist .

Statistics

For all statistical analyses, confirm that the following items are present in the figure legend, table legend, main text, or Methods section.

n/a	Confirmed	
☐	☒	The exact sample size (*n*) for each experimental group/condition, given as a discrete number and unit of measurement
☐	☒	A statement on whether measurements were taken from distinct samples or whether the same sample was measured repeatedly
☐	☒	The statistical test(s) used AND whether they are one- or two-sided<br>*Only common tests should be described solely by name; describe more complex techniques in the Methods section.*
☒	☐	A description of all covariates tested
☐	☒	A description of any assumptions or corrections, such as tests of normality and adjustment for multiple comparisons
☐	☒	A full description of the statistical parameters including central tendency (e.g. means) or other basic estimates (e.g. regression coefficient) AND variation (e.g. standard deviation) or associated estimates of uncertainty (e.g. confidence intervals)
☐	☒	For null hypothesis testing, the test statistic (e.g. *F*, *t*, *r*) with confidence intervals, effect sizes, degrees of freedom and *P* value noted<br>*Give P values as exact values whenever suitable.*
☒	☐	For Bayesian analysis, information on the choice of priors and Markov chain Monte Carlo settings
☒	☐	For hierarchical and complex designs, identification of the appropriate level for tests and full reporting of outcomes
☒	☐	Estimates of effect sizes (e.g. Cohen's *d*, Pearson's *r*), indicating how they were calculated

Our web collection on statistics for biologists contains articles on many of the points above.

Software and code

Policy information about availability of computer code

Data collection	No code were used in data collection.
Data analysis	Flow cytometry data were analyzed using FlowJo 10.5.2 software (TreeStar Inc.) and BD FACSDiva 8.0.1 software (BD Biosciences). Confocal microscopy images were analysed with Zen v1.1.2.0. Bioinformatics analysis of RNAseq data were performed using the RNA-seq pipeline from Sequana [https://pypi.org/project/sequana-rnaseq/]. Reads were cleaned of adapter sequences and low-quality sequences using cutadapt version 1.11. Bowtie version 0.12.7 with default parameters, was used for alignment on the reference genome. Genes were counted using featureCounts version 1.4.6-p3 from Subreads package (parameters: -t gene -g ID -s 1). Count data were analyzed using R version 3.4.1 (doi: 10.1093/bioinformatics/btt656) with Bioconductor package DESeq2 version 1.16 and EnrichmentBrowser R package version 2.14.3 [https://www.R-project.org/]. Curve fitting and statistical analyses were performed with GraphPad Prism versions 4.03 or 8.3.1., GraphPad InStat v3.10 (GraphPad Software), and JMP Pro version 13.1.0.

For manuscripts utilizing custom algorithms or software that are central to the research but not yet described in published literature, software must be made available to editors/reviewers. We strongly encourage code deposition in a community repository (e.g. GitHub). See the Nature Research guidelines for submitting code & software for further information.

Data

Policy information about availability of data

All manuscripts must include a data availability statement. This statement should provide the following information, where applicable:

- Accession codes, unique identifiers, or web links for publicly available datasets
- A list of figures that have associated raw data
- A description of any restrictions on data availability

The source data underlying main Figures are provided as a Source Data file. RNA-seq data reported in this study have been deposited at Gene Expression Omnibus [https://www.ncbi.nlm.nih.gov/geo/] under accession number GSE139659. GenBank [https://www.ncbi.nlm.nih.gov/nuccore/CP000253.1], KEGG orthology [https://www.genome.jp/kegg-bin/get_htext?sao00001.keg] and KEGG pathway [https://www.genome.jp/kegg-bin/show_organism?menu_type=pathway_maps&org=sao]

databases were used for gene description.

Field-specific reporting

Please select the one below that is the best fit for your research. If you are not sure, read the appropriate sections before making your selection.

- [x] Life sciences
- [ ] Behavioural & social sciences
- [ ] Ecological, evolutionary & environmental sciences

For a reference copy of the document with all sections, see nature.com/documents/nr-reporting-summary-flat.pdf

Life sciences study design

All studies must disclose on these points even when the disclosure is negative.

Sample size	No statistical pre-determination of sample size was applied. Sample sizes were defined based on prior experience with similar types of experiments showing that performing experiments at least three times with at least three replicates ensures reproducibility.
Data exclusions	No data were excluded from the analyses.
Replication	All experiments were performed with at least three independent replications, each of them performed in triplicates, by multiple experimenters to ensure reproducibility.
Randomization	Experiments were carried out on cell cultures obtained from a single subculture within each individual experiment. All plates could thus be considered as sharing the same characteristics and randomly assigned to the different experimental groups.
Blinding	Investigators were not blinded, but RNA-seq analyses, RT-PCR and intracellular infections and flow cytometry studies were performed independently by different investigators who were not aware of the results obtained by the others before putting all the data together.

Reporting for specific materials, systems and methods

We require information from authors about some types of materials, experimental systems and methods used in many studies. Here, indicate whether each material, system or method listed is relevant to your study. If you are not sure if a list item applies to your research, read the appropriate section before selecting a response.

Materials & experimental systems

n/a	Involved in the study	
x		Antibodies
	x	Eukaryotic cell lines
x		Palaeontology
x		Animals and other organisms
	x	Human research participants
x		Clinical data

Methods

n/a	Involved in the study	
x		ChIP-seq
	x	Flow cytometry
x		MRI-based neuroimaging

Eukaryotic cell lines

Policy information about cell lines

Cell line source(s)	Murine J774A.1 macrophages were obtained from Sandoz Forschung Laboratories. Human THP-1 monocytes (ATCC TIB-202), Human A549 (ATCC CCL-185) and MCF7 (ATCC HTB-22) epithelial cells were obtained from ATCC. Human MG63 osteoblastic cells were obtained from LGC Standards. Human monocytes from peripheral blood and primary keratinocytes were obtained from healthy donors.
Authentication	Cell lines were authenticated by their providers. Their characteristics and specifications are presented in details of the website of the corresponding providers.
Mycoplasma contamination	Absence of contamination by Mycoplasma is routinely done in our laboratory.
Commonly misidentified lines (See ICLAC register)	None applicable (none of the cell line used is commonly reported as misidentified).

Human research participants

Policy information about studies involving human research participants

Population characteristics	Human blood was collected in Croix-Rouge de Belgique centers, from healthy volunteers who gave written informed consent, in accordance with procedures of Service Francophone du Sang de la Croix–Rouge de Belgique.
Recruitment	Volunteers coming to give their blood at the Croix Rouge de Belgique (red Cross) and having accepted a use for research purposes
Ethics oversight	Experiments on blood material were performed in strict accordance with governmental and European legislation relative to blood, cell and tissues-related activities, and were approved by the ethical committee Comité d'Ethique Hospitalo-Facultaire Saint-Luc (CEHF Saint-Luc ; permit no. B403201730810).

Note that full information on the approval of the study protocol must also be provided in the manuscript.

Flow Cytometry

Plots

Confirm that:

- [x] The axis labels state the marker and fluorochrome used (e.g. CD4-FITC).
- [x] The axis scales are clearly visible. Include numbers along axes only for bottom left plot of group (a 'group' is an analysis of identical markers).
- [x] All plots are contour plots with outliers or pseudocolor plots.
- [x] A numerical value for number of cells or percentage (with statistics) is provided.

Methodology

Sample preparation	GFP-expressing S. aureus isolated from broth cultures or from cells were resuspended in filtered PBS, stained with 10 µg/mL propidium iodide, and analyzed for GFP signal intensities (FITC channel, medium flow rate).
Instrument	FACSVerse and FACSAria III cytometers (BD Biosciences).
Software	FlowJo 10.5.2 (TreeStar Inc.) and BD FACSDiva 8.0.1 (BD Biosciences).
Cell population abundance	All analyzed populations contained at least 5000 events. Post-sort fractions purity was determined with FlowJo 10.5.2 (TreeStar Inc.) and BD FACSDiva 8.0.1 (BD Biosciences) softwares and confirmed by cfu counting.
Gating strategy	Forward-scatter width (FCS-W) versus forward-scatter area (FSC-A), and side-scatter width (SSC-W) versus side-scatter area (SSC-A) were used to gate out damaged or multiplet cells. Of those, propidium iodide-positive bacteria were gated out.

- [x] Tick this box to confirm that a figure exemplifying the gating strategy is provided in the Supplementary Information.
